# The measurement postulates of quantum mechanics are operationally redundant

**DOI:** 10.1038/s41467-019-09348-x

**Published:** 2019-03-25

**Authors:** Lluís Masanes, Thomas D. Galley, Markus P. Müller

**Affiliations:** 1 Department of Physics and Astronomy, University College London, Gower Street, London, WC1E 6BT UK; 2 Perimeter Institute for Theoretical Physics, Waterloo, ON N2L 2Y5 Canada; 3 Institute for Quantum Optics and Quantum Information, Austrian Academy of Sciences, Boltzmanngasse 3, A-1090 Vienna, Austria

## Abstract

Understanding the core content of quantum mechanics requires us to disentangle the hidden logical relationships between the postulates of this theory. Here we show that the mathematical structure of quantum measurements, the formula for assigning outcome probabilities (Born’s rule) and the post-measurement state-update rule, can be deduced from the other quantum postulates, often referred to as “unitary quantum mechanics”, and the assumption that ensembles on finite-dimensional Hilbert spaces are characterized by finitely many parameters. This is achieved by taking an operational approach to physical theories, and using the fact that the manner in which a physical system is partitioned into subsystems is a subjective choice of the observer, and hence should not affect the predictions of the theory. In contrast to other approaches, our result does not assume that measurements are related to operators or bases, it does not rely on the universality of quantum mechanics, and it is independent of the interpretation of probability.

## Abstract (web summary)

The mathematical structure of quantum measurements and the Born rule are usually imposed as axioms; here, the authors show instead that they are the only possible measurement postulates, if we require that arbitrary partitioning of systems does not change the theory’s predictions.

## Introduction

What sometimes is postulated as a fundamental law of physics is later on understood as a consequence of more fundamental principles. An example of this historical pattern is the rebranding of the symmetrization postulate as the spin-statistics theorem^1^. Another example, according to some authors, is the Born rule, the formula that assigns probabilities to quantum measurements. The Born rule has been derived within the framework of quantum logic^2–5^, taking an operational approach^6–9^, and using other methods^10–14^. But all these derivations assume, among other things, the mathematical structure of quantum measurements, that is, the correspondence between measurements and orthonormal bases, or more generally, positive-operator valued measures^15,16^.

Taking one step further, the structure of measurements together with the Born rule can be jointly derived within the many-worlds interpretation of quantum mechanics (QM)^17,18^ and the framework of entanglement-assisted invariance^19–22^. But these derivations involve controversial uses of probability in deterministic multiverse scenarios, which have been criticized by a number of authors^21–31^. Also, these frameworks require the universality of QM, meaning that the measurement apparatus and/or the observer has to be included in the quantum description of the measuring process. While this is a meaningful assumption, it is interesting to see that it is not necessary, as proven in the present article.

In this work we take an operational approach, with the notions of measurement and outcome probability being primitive elements of the theory, but without imposing any particular structure on them. We use the fact that the subjective choices in the description of a physical setup in terms of operational primitives must not affect the predictions of the theory. For example, deciding to describe a tripartite system A·B·C as either the bipartite system AB·C or as A·BC must not modify the outcome probabilities. Using these constraints we characterize all possible alternatives to the mathematical structure of quantum measurements and the Born rule, and we prove that there is no such alternative to the standard measurement postulates. This theorem has simple and precise premises, it does not require unconventional uses of probability theory, and it is independent of the interpretation of probability. A further interesting consequence of this theorem is that the post-measurement state-update rule must necessarily be that of QM.

## Results

### The standard postulates of QM

Before presenting the main result we prepare the stage appropriately. This involves reviewing some of the postulates of QM, reconstructing the structure of mixed states from them, and introducing a general characterization of measurements that is independent of their mathematical structure.

*Postulate (states)*. To every physical system there corresponds a complex and separable Hilbert space ${\Bbb C}^d$, and the pure states of the system are the rays $\psi \in {\mathrm{P}}{\Bbb C}^d$.

It will be convenient to use the notation ${\Bbb C}^d$ both for Hilbert spaces of finite dimension *d*, and also for countably infinite-dimensional Hilbert spaces which we denote by ${\Bbb C}^\infty$. This notation is justified, since all countably infinite-dimensional Hilbert spaces are isomorphic^32^. Analogously we use *U*(∞) to denote the unitary transformations of ${\Bbb C}^\infty$. In this document we represent states (rays) by normalized vectors $\psi \in {\Bbb C}^d$.

*Postulate (transformations)*. The reversible transformations (for example, possible time evolutions) of pure states of ${\Bbb C}^d$ are the unitary transformations $\psi \mapsto U\psi$ with *U* ∈ U(*d*).

*Postulate (composite systems)*. The joint pure states of systems ${\Bbb C}^a$ and ${\Bbb C}^b$ are the rays of the tensor-product Hilbert space ${\Bbb C}^a \otimes {\Bbb C}^b$.

*Postulate (measurement)*. Each measurement outcome of system ${\Bbb C}^d$ is represented by a linear operator *Q* on ${\Bbb C}^d$ satisfying 0 ≤ *Q* ≤ ${\Bbb I}$, where ${\Bbb I}$ is the identity. The probability of outcome $Q$ on state $\psi \in {\Bbb C}^d$ is

1 $$P(Q|\psi ) = \langle \psi |Q|\psi \rangle \;.$$

A (full) measurement is represented by the operators corresponding to its outcomes $Q_1 ,..., Q_n$, which must satisfy the normalization condition $\mathop {\sum}\limits_{i = 1}^n {Q_i} = {\mathbb I}$.

The more traditional formulation of the measurement postulate in terms of (not necessarily positive) Hermitian operators is equivalent to the above. But we have chosen the above form because it is closer to the formalism used in the presentation of our results.

*Postulate (post-measurement state-update)*. Each outcome is represented by a completely-positive linear map Λ related to the operator $Q$ via

2 $${\mathrm{tr}}\Lambda (|\psi \rangle \langle \psi |) = \langle \psi |Q|\psi \rangle \;,$$

for all *ψ*. The post-measurement state after outcome Λ is

3 $$\rho = \frac{{\Lambda (|\psi \rangle \langle \psi |)}}{{{\mathrm{tr}}\Lambda (|\psi \rangle \langle \psi |)}}\;.$$

A (full) measurement is represented by the maps corresponding to its outcomes Λ_1_, …, Λ_*n*_ whose sum $\mathop {\sum}\limits_{i = 1}^n {\Lambda _i}$ is trace-preserving.

If the measurement is repeatable and minimally disturbing^33,34^ then $Q_1 ,..., Q_n$ are projectors and the above maps are of the form Λ_*i*_(*ρ*) = $Q_i  \rho Q_i$, which is the standard textbook “projection postulate”. Below we prove that the “measurement” and “post-measurement state-update” postulates are a consequence of the first three postulates.

### The structure of mixed states

Mixed states are not mentioned in the standard postulates of QM, but their structure follows straightaway from the measurement postulate (1). Recall that a mixed state is an equivalence class of indistinguishable ensembles, and an ensemble (*ψ*_*r*_, *p*_*r*_) is a probability distribution over pure states. Note that the notion of distinguishability depends on what the measurements are. For the particular case of quantum measurements (1), the probability of outcome $Q$ when a source prepares state *ψ*_*r*_ with probability *p*_*r*_ is

4 $$P(Q|(\psi _r,p_r)) = \mathop {\sum}\limits_r {p_r} P(Q|\psi _r) = {\mathrm{tr}}(Q\rho )\;,$$

where we define the density matrix

5 $$\rho = \mathop {\sum}\limits_r {p_r} |\psi _r\rangle \langle \psi _r|\;.$$

This matrix contains all the statistical information of the ensemble. Therefore, two ensembles with the same density matrix are indistinguishable.

The important message from the above is that a different measurement postulate would give different equivalence classes of ensembles, and hence, a different set of mixed states. Thus, in proving our main result, we will not assume that mixed states are of the form (5). An example of mixed states for a non-quantum measurement postulate is described in the section “Non-quantum measurement postulate violating associativity”. A full classification of the sets of mixed states for non-quantum measurement postulates is given in Supplementary Note 1.

### Formalism for any alternative measurement postulate

Before proving that the only possible measurement postulate is that of QM, we have to articulate what “a measurement postulate” is in general. In order to do so, we introduce a theory-independent characterization of measurements for single and multipartite systems. This is based on the concept of outcome probability function (OPF), introduced in^35^ and defined next.

*Definition (OPF)*. Each measurement outcome that can be observed on system ${\Bbb C}^d$ is represented by the function $f:{\mathrm{P}}{\Bbb C}^d \to [0,1]$ being its corresponding probability $f(\psi)=P(f|\psi)$ for each pure state $\psi \in {\mathrm{P}}{\Bbb C}^d$; and we denote by ${\cal F}_d$ the complete set of OPFs of system ${\Bbb C}^d$. Completeness is defined below as the closure of ${\cal F}_d$ under various operations.

If instead of a single outcome we want to specify a full measurement with, say, *n* outcomes, we provide the OPFs $f_1 ,..., f_n$ corresponding to each outcome; which must satisfy the normalization condition

6 $$\mathop {\sum}\limits_{i = 1}^n {f_i} (\psi ) = 1\;,$$

for all states *ψ*.

It is important to note that this mathematical description of measurements is independent of the underlying interpretation of probability: all we are assuming is that there exist experiments which yield definite outcomes (possibly relative to a given agent who uses this formalism), and that it makes sense to assign probabilities to these outcomes. For example, we could interpret them as Bayesian probabilities of a physicist who bets on future outcomes of experiments; or as limiting frequencies of a large number of repetitions of the same experiment, approximating empirical data. Whenever we have an experiment of that kind, the corresponding probabilities (whatever they mean) will be determined by a collection of OPFs.

The completeness of the set of OPFs ${\cal F}_d$ consists of the following three properties:

*Property 1 (*${\cal F}_d$ *is closed under taking mixtures):* Suppose that the random variable *x* with probability *p*_*x*_ determines which 2-outcome measurement $f_1^x,f_2^x \in {\cal F}_d$ we implement, and later on we forget the value of *x*. Then the probability of outcome 1 for this “averaged” measurement is

7 $$\mathop {\sum}\limits_x {p_x} {\mkern 1mu} f_1^x \in {\cal F}_d\;,$$

which must be a valid OPF. Therefore, mixtures of OPFs are OPFs.

*Property 2 (*${\cal F}_d$ *is closed under composition with unitaries):* We can always perform a transformation *U* ∈ U(*d*) before a measurement $f \in {\cal F}_d$, effectively implementing the measurement

8 $$f \circ U \in {\cal F}_d\;,$$

which then must be a valid OPF. Note that here we are not saying that all unitaries can be physically implemented, but only that the formalism must in principle include them.

*Property 3 (*${\cal F}_d$ *is closed under systems composition):* Since ${\cal F}_d$ is complete, it also includes the measurements that appear in the description of ${\Bbb C}^d$ as part of the larger system ${\Bbb C}^d \otimes {\Bbb C}^b \cong {\Bbb C}^{db}$, for any background system ${\Bbb C}^b$. Formally, for each background state $\varphi \in {\Bbb C}^b$ and global OPF $g \in {\cal F}_{db}$ there is local OPF $f_{\varphi ,g} \in {\cal F}_d$ which represents the same measurement outcome

9 $$f_{\varphi ,g}(\psi ) = g(\psi \otimes \varphi )\;,$$

$$\psi \in {\mathrm{P}}{\Bbb C}^d\;.$$

Next we consider local measurements in multipartite systems. In order to do so, it is useful to recall that the observer always has the option of describing a systems ${\Bbb C}^a$ as part of a larger system ${\Bbb C}^a \otimes {\Bbb C}^b$, without this affecting the predictions of the theory. In order to do so, the observer needs to know how to represent the OPFs of the small system ${\cal F}_a$ as OPFs of the larger system ${\cal F}_{ab}$. This information is contained in the star product, defined in what follows.

*Definition (*⋆*-product)*. Any pair of local OPFs, $f \in {\cal F}_a$ and $g \in {\cal F}_b$, is represented as a global OPF $(f \star g) \in {\mathrm{ }}{\cal F}_{ab}$ via the star product $\star :{\cal F}_a \times {\cal F}_b \to {\cal F}_{ab}$, which satisfies

10 $$(f \star g)(\psi \otimes \varphi ) = {\mathrm{ }}f(\psi ){\mkern 1mu} g(\varphi )\;,$$

for all $\psi \in {\mathrm{P}}{\Bbb C}^a$ and $\varphi \in {\mathrm{P}}{\Bbb C}^b$. This product must be defined for any pair of (complex and separable) Hilbert spaces ${\Bbb C}^a$ and ${\Bbb C}^b$.

In other words, the ⋆-product represents bi-local measurements, which in QM are represented by the tensor product in the space of Hermitian matrices.

Since the option of describing system ${\Bbb C}^a$ as part of a larger system ${\Bbb C}^a \otimes {\Bbb C}^b$ is a subjective choice that must not affect the predictions of the theory, the embedding of ${\cal F}_a$ into ${\cal F}_{ab}$ provided by the ⋆-product must preserve the structure of ${\cal F}_a$. This includes the mixing (convex) structure

11 $$\left( {\mathop {\sum}\limits_x {p_x} f^x} \right) \star g = \mathop {\sum}\limits_x {p_x} \left( {f^x \star g} \right)\;,$$

as well as the U(*d*) action

12 $$\left( {f \circ U} \right) \star g = \left( {f \star g} \right) \circ (U \otimes {\mathbb{I}}_{b})\;.$$

And likewise for the other party ${\cal F}_b$. The ⋆-product must also preserve probability, in the sense that if $\{ f_i\} \subseteq {\cal F}_a$ and $\{ g_j\} \subseteq {\cal F}_b$ are full measurements satisfying the normalization condition (6) then we must have

13 $$\left[ {(\mathop {\sum}\limits_i {f_i} ) \star (\mathop {\sum}\limits_j {g_i} )} \right](\psi ) = 1\;,$$

for all rays *ψ* of ${\Bbb C}^a \otimes {\Bbb C}^b$.

Pushing the same philosophy further, the observer has the option of describing the tripartite system ${\Bbb C}^a \otimes {\Bbb C}^b \otimes {\Bbb C}^c$ as the bipartite system ${\Bbb C}^a \otimes [{\Bbb C}^b \otimes {\Bbb C}^c]$ or the bipartite system $[{\Bbb C}^a \otimes {\Bbb C}^b] \otimes {\Bbb C}^c$, without this affecting the probabilities predicted by the theory. This translates to the ⋆-product being associative

14 $$f \star \left( {g \star h} \right) = (f \star g) \star h$$

That is, the probability of outcome $f \star g \star h$ is independent of how we choose to partition the global system into subsystems. As we show below, this property will be crucial to recover the standard measurement postulates of QM.

### The measurement theorem

Before stating the main result of this work, we specify what should be the content of any alternative measurement postulate, and state an operationally-meaningful assumption that is necessary to prove our theorem.

*Definition (measurement postulate)*. This is a family of OPF sets ${\cal F}_2,{\cal F}_3,{\cal F}_4, \ldots$ and ${\cal F}_\infty$ equipped with a ⋆-product ${\cal F}_a \times {\cal F}_b \to {\cal F}_{ab}$ satisfying conditions (7–14).

In addition to the above, a measurement postulate could provide restrictions on which OPFs can be part of the same measurement (beyond the normalization condition). However, such rules would not affect our results.

*Assumption (possibility of state estimation)*. Each finite-dimensional system ${\Bbb C}^d$ has a finite list of outcomes $f^1, \ldots ,f^k \in {\cal F}_d$ such that knowing their value on any ensemble (*ψ*_*r*_, *p*_*r*_) allows us to determine the value of any other OPF $g \in {\cal F}_d$ on the ensemble (*ψ*_*r*_, *p*_*r*_).

It is important to emphasize that $f^1 ,..., f^k$ need not be outcomes of the same measurement; and also, this list need not be unique. For example, in the case of QM, we can specify the state of a spin-$\frac{1}{2}$ particle with the probabilities of outcome “up” in any three linearly independent directions. Also in QM, we have *k* = *d*^2^ − 1; but here we are not assuming any particular relation between *d* and *k*. Now it is time to state the main result of this work, which essentially tells us that the only possible measurement postulates are the quantum ones.

*Theorem (measurement)*. The only measurement postulate satisfying the “possibility of state estimation” has OPFs and ⋆-product of the form

15 $$f(\varphi ) = \langle \varphi |F|\varphi \rangle \;,$$

16 $$(f \star g)(\psi ) = \langle \psi |F \otimes G|\psi \rangle \;,$$

for all $\varphi \in {\Bbb C}^a$ and $\psi \in {\Bbb C}^a \otimes {\Bbb C}^b$, where the ${\Bbb C}^a$-operator *F* satisfies $0 \leq F \leq {\Bbb I}$, and analogously for *G*.

The methods section provides a summary of the ideas and techniques used in the proof of this theorem. Full detail can be found in  Supplementary Note 3 and Supplementary Note 4.

### The post-measurement state-update rule

At first sight, the above theorem says nothing about the post-measurement state-update rule. But actually, it is well-known^36^ that the only possible state-update rule that is compatible with the probability rule implied by the theorem (15–16) is the one stated above in postulate “post-measurement state-update rule”. We include a self-contained proof of the above in Supplementary Note 5.

### Non-quantum measurement postulate violating associativity

In this section we present an example of alternative measurement postulate, which shows that it is possible to bypass the measurement theorem if we give up the associativity condition (14). It also illustrates how a different choice of measurement postulate produces a different set of mixed states.

*Definition (non-quantum measurement postulate)*. An *n*-outcome measurement on ${\Bbb C}^a$ is characterized by *n* Hermitian operators *F*_*i*_ acting on ${\Bbb C}^a \otimes {\Bbb C}^a$ and satisfying $0 \le F_i \le P_ + ^a$ and

17 $$\mathop {\sum}\limits_{i = 1}^n {F_i} = P_ + ^a\;,$$

where $P_ + ^a$ is the projector onto the symmetric subspace of ${\Bbb C}^a \otimes {\Bbb C}^a$. The probability of outcome *i* on the (normalized) state $\varphi \in {\Bbb C}^a$ is given by

18 $$f_i(\varphi ) = {\mathrm{ tr}}\left( {F_i|\varphi \rangle \langle \varphi |^{ \otimes 2}} \right);$$

and the ⋆-product of two OPFs $f \in {\cal F}_a$ and $g \in {\cal F}_b$ of the form (18) is defined as

$$(f \star g)(\psi ) = {\mathrm{tr}}\left[ {\left( {F \otimes G + \frac{{{\mathrm{tr}}{\mkern 1mu} F}}{{{\mathrm{tr}}P_ + ^a}}P_ - ^a \otimes \frac{{{\mathrm{tr}}{\mkern 1mu} G}}{{{\mathrm{tr}}P_ + ^b}}P_ - ^b} \right)|\psi \rangle \langle \psi |^{ \otimes 2}} \right],$$

for any normalized $\psi \in {\mathrm{ }}{\Bbb C}^a \otimes {\Bbb C}^b$.

This alternative theory violates the principles of “local tomography”^37^ and “purification”^38^. This and other exotic properties of this theory are analyzed in detail in previous work^35,39^. Also, the validity of marginal and conditional states imposes additional constraints on the matrices *F* which are also worked out in^39^. It is easy to check that the above definition satisfies conditions (7–13) and violates associativity (14). Therefore, this provides a perfectly valid toy theory of systems that encompass either one or two components, but not more.

As we have mentioned above, the structure of the mixed states depends on the measurement postulate. Here, the mixed state corresponding to ensemble (*ψ*_*r*_, *p*_*r*_) is

19 $$\omega = \mathop {\sum}\limits_r {p_r} |\psi _r\rangle \langle \psi _r|^{ \otimes 2}\;.$$

Another non-quantum property of this toy theory is that the uniform ensembles corresponding to two different orthonormal bases, {*φ*_*i*_} and {*ψ*_*i*_} are distinguishable

20 $$\mathop {\sum}\limits_i {\frac{1}{d}} |\varphi _i\rangle \langle \varphi _i|^{ \otimes 2} \ne \mathop {\sum}\limits_i {\frac{1}{d}} {\mkern 1mu} |\psi _i\rangle \langle \psi _i|^{ \otimes 2}\;.$$

### Gleason’s theorem and non-contextuality

As mentioned in the introduction, Gleason’s theorem and many other derivations of the Born rule^2–8,10,12^ assume the structure of quantum measurements; that is, the correspondence between measurements and orthonormal bases {*φ*_*i*_}, or more generally, positive-operator valued measures^16^. But in addition to this, they assume that the probability of an outcome *φ*_*i*_ does not depend on the measurement (basis) it belongs to. Note that this type of “non-contextuality” is already part of the content of Born’s rule.

To show that this “non-contextuality” assumption is by no means necessary, we review an alternative to the Born rule, presented in ref. ^40^, which does not satisfy it. In this toy theory, we also have that measurements are associated to orthonormal bases {*φ*_*i*_} and each outcome corresponds to an element *φ*_*i*_ of the basis. Then, the probability of outcome *φ*_*i*_ on state *ψ* is given by

21 $$P(\varphi _i|\psi ) = \frac{{|\langle \varphi _i|\psi \rangle |^4}}{{\mathop {\sum}\limits_j | \langle \varphi _j|\psi \rangle |^4}}\;.$$

Since this example does not meet the premises of Gleason’s theorem (the denominator depends not only on *φ*_*i*_ but also on the rest of the basis), there is no contradiction in that it violates its conclusion.

We stress that our results, unlike previous contributions^2–8,10,12^, do not assume this type of non-contextuality. In particular, our OPF framework perfectly accommodates the above example (21) with $f_i (\psi)=P({\varphi}_i|{\psi})$. This example however does not meet the “possibility of state estimation” assumption, and hence is excluded by the main theorem of this paper.

In the Supplementary Discussion we discuss publications^13^ and^41^ in relation to the theorem presented in this paper.

## Discussion

It may seem that conditions (7–14) are a lot of assumptions to claim that we derive the measurement postulates from the non-measurement ones.

But from the operational point of view, these conditions constitute the very definition of measurement, single and multi-partite physical system. In other words, specifying what we mean by “measurement” is in a different category than stating that measurements are characterized by operators acting on a Hilbert space. Analogously, the rules of probability calculus or the axioms of the real numbers are not explicitly included in the postulates of QM.

Note that our results also apply to indistinguishable particles (bosons and fermions), as long as we interpret the tensor product not as a composition of particles, but of the corresponding modes.

It is rather remarkable that none of the three measurement postulates (structure, probabilities and state-update) can be modified without having to redesign the whole theory. In particular, the probability rule is deeply ingrained in the main structures of the theory. This fact shows that one need not appeal to any supplementary principles beyond operational primitives to derive the Born rule, nor do we need to make any assumptions about the structure of measurements, unlike previous work^6,10,12,18,19,40^. Finally, having cleared up unnecessary postulates in the formulation of QM, we find ourselves closer to its core message.

## Methods

This brief section provides a bird’s eye view of the proof of the measurement theorem of Supplementary Note 3 and Supplementary Note 4. The argument starts by embedding the OPF set ${\cal F}_d$ into a complex vector space so that physical mixtures (7) can be represented by certain linear combinations. Second, the “possibility of state estimation” assumption implies that, whenever *d* is finite, this embedding vector space is finite-dimensional. This translates the U(*d*) action (8) on the set ${\cal F}_d$ to a linear representation; and once in the land of U(*d*) representations we have a good map of the territory.

Third, the fact that the argument of the functions in ${\cal F}_d$ is a ray (not a vector) imposes a strong restriction to the above-mentioned U(*d*) representation. All these restricted representations were classified by some of the authors in ref. ^35^. This amounts to a classification of all alternatives to the measurement postulate for single systems, that is, when the consistency constraints related to composite systems (9–14) are ignored. The next steps take composition into account.

Fourth, “closedness under system composition” (9) implies that all OPFs $f \in {\cal F}_d$ are of the form

22 $$f(\varphi ) = {\mathrm{tr}}\left( {F|\varphi \rangle \langle \varphi |^{ \otimes n}} \right)\;,$$

where *n* is a fixed positive integer, as shown in Supplementary Note 2. Recall that the case *n* = 1 is QM and the case *n* = 2 has been studied above. In the final step, the representation theory of the unitary group is exploited to prove that, whenever *n* ≥ 2, it is impossible to define a star product of functions (22) satisfying associativity (14). This implies that only the quantum case (*n* = 1) fulfils all the required constraints (7–14).

## Supplementary information

Supplementary Information

## Data Availability

No data sets were generated or analyzed during the current study.
