# Supplementary material for: The measurement postulates of quantum mechanics are operationally redundant
Source: Nat Commun. 2019 Mar 25;10:1361. doi: 10.1038/s41467-019-09348-x (PMC6434053; doi:10.1038/s41467-019-09348-x)
Supplement: Supplementary file 1 — Supplementary Information [file 41467_2019_9348_MOESM1_ESM.pdf]

The Measurement Postulates of Quantum Mechanics are Operationally Redundant - Supplementary Information

Masanes et al.

Supplementary note 1: Alternative measurement postulates for single systems

In this section we classify all alternative measurement postulates for the case of finite-dimensional single systems, that is, when the constraints associated to the composition of systems (the star product) are ignored. These results build up on the previous work [1] by two of us.

In this section we only consider finite-dimensional Hilbert spaces $\mathbb{C}^d$ with d a positive integer. In this case we have $\mathrm{U}(d) \cong \mathrm{SU}(d) \times \mathrm{U}(1)$; and since $\mathrm{U}(1)$ has a trivial action on rays, we only consider $\mathrm{SU}(d)$. Later on, when addressing the infinite-dimensional case $d = \infty$, we will work with $\mathrm{U}(d)$, since the condition $\det U = 1$ is not well-defined when $d = \infty$.

Structure of measurements and mixed states

Definition 1. $\mathcal{F}_d$ is a set of functions $\mathbf{f} : \mathrm{P}\mathbb{C}^d \to [0,1]$ which is closed under composition with unitaries $U \in \mathrm{SU}(d)$

$$U : \mathbf{f} \mapsto (\mathbf{f} \circ U) \ , \tag{1}$$

closed under convex combinations

$$\sum_x p_x \, \mathbf{f}^x \in \mathcal{F}_d \ , \tag{2}$$

and that contains the unit and the zero functions, respectively $\mathbf{u}(\psi) = 1$ and $\mathbf{0}(\psi) = 0$, for all $\psi \in \mathrm{P}\mathbb{C}^d$.

The unit function $\mathbf{u}$ represents an outcome that happens with probability one. For example, such unit-probability outcome can be the event corresponding to all outcomes of the measurement $\{\mathbf{f}_i\}$, which by normalization satisfy

$$\sum_i \mathbf{f}_i = \mathbf{u} \ . \tag{3}$$

Analogously, the zero function $\mathbf{0}$ represents a formal outcome that has zero probability irrespectively of the state.

For what comes below, it is convenient to consider the set $\mathcal{F}_d$ as embedded in the complex vector space $\mathbb{C}\mathcal{F}_d$ generated by itself. The fact that the group action (1) commutes with the mixing operation (2)

$$\left(\textstyle\sum_x p_x \, \mathbf{f}^x\right) \circ U = \textstyle\sum_x p_x (\mathbf{f}^x \circ U) \ , \tag{4}$$

can be extended to arbitrary linear combinations in $\mathbb{C}\mathcal{F}_d$, providing a complex, linear representation of $\mathrm{SU}(d)$. While only the elements of $\mathcal{F}_d$ are outcome probability functions (OPFs), any element of $\mathbb{C}\mathcal{F}_d$ can be interpreted as the expectation value of an observable with complex outcome labels, in analogy to the algebra of observables in QM. While in QM the space $\mathbb{C}\mathcal{F}_d$ has dimension d^2, here we leave the dimension unconstrained. However, in what follows, we show that the "possibility of state estimation" assumption implies that the linear space $\mathbb{C}\mathcal{F}_d$ is finite-dimensional. But before this, we recall that the probability of outcome $\mathbf{f} \in \mathcal{F}_d$ on an ensemble (ψ_r, p_r) is given by

$$\mathbf{f}[(\psi_r, p_r)] = \sum_r p_r \, \mathbf{f}[\psi_r] \ . \tag{5}$$

The above follows from the rules of probability calculus.

Lemma 2. Suppose that the values of the outcomes $\mathbf{f}^1, \ldots, \mathbf{f}^k \in \mathcal{F}_d$ on any given ensemble (ψ_r, p_r) determine the value of any other outcome $\mathbf{g} \in \mathcal{F}_d$ on that ensemble (ψ_r, p_r). Then the functions $\{\mathbf{f}^1, \ldots, \mathbf{f}^k, \mathbf{u}\}$ span the linear space $\mathbb{C}\mathcal{F}_d$.

In other words, knowing the numbers $\mathbf{f}^1[(\psi_r, p_r)]$, $\ldots$, $\mathbf{f}^k[(\psi_r, p_r)]$ allows us to determine the number $\mathbf{g}[(\psi_r, p_r)]$ without knowing the ensemble (ψ_r, p_r). That is, the latter is some function of the former.

Proof. Once the OPFs $\mathbf{f}^1, \ldots, \mathbf{f}^k \in \mathcal{F}_d$ are given we can define the convex set

$$\mathcal{S}_d = \mathrm{conv}\left\{ \left[\mathbf{f}^1(\psi), \ldots, \mathbf{f}^k(\psi)\right] \ | \ \psi \in \mathrm{P}\mathbb{C}^d \right\} \subseteq \mathbb{R}^k \ . \tag{6}$$

Next we note that, the fact that the values of $\mathbf{f}^1, \ldots, \mathbf{f}^k$ determine the value of $\mathbf{g}$ on any ensemble (ψ_r, p_r) means that there is a function $\xi_{\mathbf{g}} : \mathcal{S}_d \to [0,1]$ such that

$$\sum_r p_r \mathbf{g}(\psi_r) = \xi_{\mathbf{g}}\left[\textstyle\sum_r p_r \mathbf{f}^1(\psi_r), \ldots, \sum_r p_r \mathbf{f}^k(\psi_r)\right] \ . \tag{7}$$

Since the above equality holds for all ensembles, it also holds for the pure states ψ_r

$$\mathbf{g}(\psi_r) = \xi_{\mathbf{g}}\big[\mathbf{f}^1(\psi_r), \ldots, \mathbf{f}^k(\psi_r)\big] \ , \tag{8}$$

for all r. Hence we have

$$\begin{aligned}\xi_{\mathbf{g}}&\left[\textstyle\sum_r p_r \mathbf{f}^1(\psi_r), \ldots, \sum_r p_r \mathbf{f}^k(\psi_r)\right] \\ &= \textstyle\sum_r p_r\, \xi_{\mathbf{g}}\big[\mathbf{f}^1(\psi_r), \ldots, \mathbf{f}^k(\psi_r)\big] \ .\end{aligned} \tag{9}$$

It follows that (9) also holds true if every appearance of ψ_r is replaced by some ensemble $(\psi_s^{(r)}, q_s^{(r)})$, where s labels the possible states and their probabilities. Since $(\mathbf{f}^1[(\psi_s^{(r)}, q_s^{(r)})], \ldots, \mathbf{f}^k[(\psi_s^{(r)}, q_s^{(r)})])$ can take all values in $\mathcal{S}_d$ by choosing the states and probabilities in a suitable way, this shows that $\xi_{\mathbf{g}}$ is convex on the full set $\mathcal{S}_d$. This implies that $\xi_{\mathbf{g}}$ can be affinely extended to all of $\mathbb{R}^k$, i.e. there is an affine function $\xi'_{\mathbf{g}} : \mathbb{R}^k \to \mathbb{R}$ which coincides with the previous function $\xi'_{\mathbf{g}} = \xi_{\mathbf{g}}$ inside the convex set $\mathcal{S}_d$. The affine nature of the function means

$$\xi'_{\mathbf{g}}(\textstyle\sum_r c_r\, \vec{x}_r) = \sum_r c_r\, \xi'_{\mathbf{g}}(\vec{x}_r) \ , \tag{10}$$

for any $c_r \in \mathbb{R}$ with $\sum_r c_r = 1$ and $\vec{x}_r \in \mathbb{R}^k$, but the coefficients c_r are not necessarily positive. Any affine function $\xi'_{\mathbf{g}} : \mathbb{R}^k \to \mathbb{R}$ can be written as

$$\xi'_{\mathbf{g}}(\vec{x}) = \vec{e}_{\mathbf{g}} \cdot \vec{x} + c_{\mathbf{g}} \ , \tag{11}$$

where $\vec{e}_{\mathbf{g}} \in \mathbb{R}^k$ and $c_{\mathbf{g}} \in \mathbb{R}$. Therefore we can write

$$\mathbf{g} = \sum_x e^x_{\mathbf{g}}\, \mathbf{f}^x + c_{\mathbf{g}}\, \mathbf{u} \ . \tag{12}$$

That is, any OPF $\mathbf{g}$ can be written as an $\mathbb{R}$-linear combination of $\{\mathbf{f}^1, \ldots, \mathbf{f}^k, \mathbf{u}\}$, as in (12). Since every element of $\mathbb{C}\mathcal{F}_d$ is a complex-linear combination of such OPFs, every such element must thus be a complex-linear combination of $\{\mathbf{f}^1, \ldots, \mathbf{f}^k, \mathbf{u}\}$. □

Corollary 3. The "possibility of state estimation" assumption implies that, for all finite d, the linear space $\mathbb{C}\mathcal{F}_d$ is finite-dimensional.

In what follows, we introduce a representation of pure states ψ that is linearly related to outcome probabilities. Because of this, this new representation encodes the equivalence relation between ensembles, and hence, the structure of mixed states arising from alternative measurement postulates.

Definition 4. For each pure state $\psi \in \mathrm{P}\mathbb{C}^d$ we define the linear form $\Omega_\psi : \mathbb{C}\mathcal{F}_d \to \mathbb{C}$ as

$$\Omega_\psi(\mathbf{f}) = \mathbf{f}(\psi) \ , \tag{13}$$

with the natural $\mathrm{SU}(d)$ action

$$U : \Omega_\psi \mapsto \Omega_{U\psi} \ . \tag{14}$$

This allows to write the probability of outcome $\mathbf{f} \in \mathcal{F}_d$ on ensemble (ψ_r, p_r),

$$P\big(\mathbf{f}\big|(\psi_r, p_r)\big) = \textstyle\sum_r p_r P(\mathbf{f}|\psi_r) = \omega(\mathbf{f}) \ , \tag{15}$$

in terms of the mixed state

$$\omega = \textstyle\sum_r p_r\, \Omega_{\psi_r} \ . \tag{16}$$

Hence, two different ensembles corresponding to the same mixed state (16) are indistinguishable. The next lemma gives us important information about the group representation $\mathbb{C}\mathcal{F}_d$.

Lemma 5. The $\mathrm{SU}(d)$ action (1) on $\mathbb{C}\mathcal{F}_d$ decomposes as

$$\mathbb{C}\mathcal{F}_d \cong \bigoplus_{j \in \mathcal{J}} \mathcal{N}_j^d \ , \tag{17}$$

where $\mathcal{N}_j^d$ are the irreducible representations defined in Lemma 7. The finite set $\mathcal{J}$ contains zero and some positive integers (with no repetitions).

Before proving the above we mention that the quantum case is $\mathcal{J} = \{0, 1\}$, and in section Non-quantum measurement postulate violating associativity of the main text the (non-quantum) case $\mathcal{J} = \{0, 1, 2\}$ is analyzed. Also, we have to mention that in this work we follow the notation of [2], where the group representations are labelled by the subspace they act on.

Proof. In this proof we establish the following four facts in the same order: (i) $\mathbb{C}\mathcal{F}_d$ decomposes into a finite sum of finite-dimensional irreducible representations (irreps), (ii) these irreps are of the type $\mathcal{N}_j^d$, (iii) there are no repetitions, (iv) $j = 0$ is always included.

Fact (i). Lemma 2 shows that the SU(d) representation $\mathbb{C}\mathcal{F}_d$ is finite-dimensional. And these can always be decomposed into finite-dimensional irreps [2]. Also, we know that each finite-dimensional irrep of SU(d) corresponds to a d-row Young diagram λ. Hence we write

$$\mathbb{C}\mathcal{F}_d \cong \bigoplus_\lambda \mathcal{V}_\lambda^d \ , \tag{18}$$

where repeated values of λ can happen.

Fact (ii). The fact that different elements of $\mathbb{C}\mathcal{F}_d$ are different functions $\mathrm{P}\mathbb{C}^d \to \mathbb{C}$ implies that the form Ω_ψ (Definition 4) has support in each sub-space of (18). Indeed, if Ω_ψ had no support in the sub-space $\mathcal{V}_\lambda^d$, then any of the elements $\mathbf{f} + \mathcal{V}_\lambda^d \subseteq \mathbb{C}\mathcal{F}_d$ would correspond to the same function.

Denote by SU(d,ψ) the subgroup of unitaries that leave the state ψ invariant $U\psi = \psi$, and note that

$$\mathrm{SU}(d,\psi) \cong \mathrm{U}(1) \times \mathrm{SU}(d-1) \ , \tag{19}$$

for any ψ. According to (14), the action of SU(d,ψ) on a subspace $\mathcal{V}_\lambda^d$ of (18) leaves the projection of Ω_ψ onto the subspace $\mathcal{V}_\lambda^d$ invariant. This implies that $\mathcal{V}_\lambda^d$ contains an invariant vector under the action of SU(d,ψ). But Lemma 1 from [1] tells us that the only SU(d) irreps with an SU(d,ψ)-invariant vector are $\mathcal{N}_j^d$ for $j = 0, 1, 2, \ldots$ Hence, all irreps $\mathcal{V}_\lambda^d$ in (18) are of the form $\mathcal{N}_j^d$. At this point it is worth mentioning that the irreps $\mathcal{N}_j^d$ are real.

In addition, Lemma 1 from [1] tells us that in each irrep $\mathcal{N}_j^d$ the SU(d,ψ)-invariant subspace has dimension one. Which fixes the projection of the linear form Ω_ψ onto each subspace of (17) up to a proportionality factor. Changing these proportionality factors modifies the structure of $\mathcal{F}_d$ by the corresponding inverse linear transformation; but the space $\mathbb{C}\mathcal{F}_d$ remains identical.

To prove Fact (iii), suppose that there are two repeated irreps in (17). We can write the isomorphism

$$\mathcal{N}_j^d \oplus \mathcal{N}_j^d \cong \mathcal{N}_j^d \otimes \mathbb{C}^2 \ , \tag{20}$$

with the understanding that the SU(d)-action in $\mathbb{C}^2$ is trivial. Next, we invoke the above-shown unicity of Ω_ψ to see that the projection of Ω_ψ onto the subspace $\mathcal{N}_j^d \otimes \mathbb{C}^2$ is of the form

$$\Omega_\psi|_{\mathcal{N}_j^d \otimes \mathbb{C}^2} = \Omega_\psi|_{\mathcal{N}_j^d} \otimes \Gamma|_{\mathbb{C}^2} \ , \tag{21}$$

where $\Gamma : \mathbb{C}^2 \to \mathbb{C}$ is a linear form that depends on the above-mentioned proportionality factors. Given Γ it possible to find two different vectors $\mathbf{v}, \mathbf{v}' \in \mathbb{C}^2$ such that $\Gamma(\mathbf{v}) = \Gamma(\mathbf{v}')$. Then, taking any $\mathbf{f} \in \mathcal{N}_j^d$ we can construct two different elements of $\mathbb{C}\mathcal{F}_d$ corresponding to the same function

$$(\mathbf{f} \otimes \mathbf{v})(\psi) = (\mathbf{f} \otimes \mathbf{v}')(\psi) \tag{22}$$

for all ψ, which is a non-sense.

To establish Fact (iv), we recall that the unit function $\mathbf{u} \in \mathcal{F}_d$ is always included (Definition 1). Since $\mathbf{u}$ is invariant under the action (1) the trivial irrep $\mathcal{N}_0^d$ must be included in the decomposition (17). Hence $0 \in \mathcal{J}$. □

The SU(d) representations $\mathcal{M}_n^d$ and $\mathcal{N}_n^d$

In this subsection we introduce two families of SU(d) representations that allow to construct all alternative measurement postulates for single systems by using (17). For this, we recall that the projector onto the symmetric subspace of $(\mathbb{C}^d)^{\otimes n}$ can be written as the average of all permutations π over n objects

$$P_+ = \frac{1}{n!} \sum_\pi \pi \ , \tag{23}$$

where π acts by permuting the n factor spaces of $(\mathbb{C}^d)^{\otimes n}$. Next we define an SU($d$) representation that sometimes is named $\mathrm{Sym}^n\mathbb{C}^d \otimes \mathrm{Sym}^n\mathbb{C}^{d*}$.

Definition 6. Let $\mathcal{M}_n^d$ be the linear space of complex matrices M acting on $(\mathbb{C}^d)^{\otimes n}$ whose support is contained in the symmetric subspace

$$P_+M = MP_+ = M \ . \tag{24}$$

And let the linear action of SU(d) on $\mathcal{M}_n^d$ be

$$M \mapsto U^{\otimes n} M U^{\otimes n\dagger} \ . \tag{25}$$

Lemma 7. The decomposition of $\mathcal{M}_n^d$ into SU(d) irreducible representations is

$$\mathcal{M}_n^d = \bigoplus_{j=0}^{n} \mathcal{N}_{j,n}^d \ , \tag{26}$$

where the subspace $\mathcal{N}_{j,n}^d$ is generated by applying the group action (25) to the element

$$N_{j,n} = P_+ \left(|0\rangle\langle 1|^{\otimes j} \otimes \mathbb{1}^{\otimes (n-j)} \right) P_+ \in \mathcal{M}_n^d \ , \tag{27}$$

where $|0\rangle, |1\rangle \in \mathbb{C}^d$ are any orthogonal pair. Also, the representation isomorphisms

$$\mathcal{N}_{j,n}^d \cong \mathcal{N}_{j,n'}^d \tag{28}$$

hold for all $n, n' \geq j$.

Isomorphism (28) allows us to use the shorthand notation $\mathcal{N}_j^d$. Also, note that $\mathcal{N}_0^d$ is the trivial irrep, generated by the element $N_{0,n} = P_+ \in \mathcal{M}_n^d$; and $\mathcal{N}_1^d$ is the adjoint (quantum) irrep.

Proof. In order to obtain the decomposition (26) it is useful to define the trace map

$$\mathrm{tr}_n : \mathcal{M}_n^d \to \mathcal{M}_{n-1}^d \ , \tag{29}$$

$$M \mapsto \mathrm{tr}_n M \ , \tag{30}$$

where tr_n denotes the trace over the nth factor in $(\mathbb{C}^d)^{\otimes n}$. Note that, by symmetry, this partial trace is independent of the choice of factor: $\mathrm{tr}_n M = \mathrm{tr}_1 M$. From now on, wherever is clear, we leave the dependence on d implicit.

Because the map (29) commutes with the SU(d) action,

$$\mathrm{tr}_n \left[U^{\otimes n} M U^{\otimes n \dagger} \right] = U^{\otimes (n-1)} \mathrm{tr}_n [M] \, U^{\otimes (n-1)\dagger} \ , \tag{31}$$

Schur's Lemma tells us that its kernel must be a subrepresentation of $\mathcal{M}_n$, which we denote by $\mathcal{N}_{n,n}$. It is proven in Lemma 23 that this representation is irreducible. Also, it is straightforward to check that the matrix $N_{n,n}$ defined in (27) is in the kernel of the map (29), that is

$$\mathrm{tr}_n N_{n,n} = 0 \ . \tag{32}$$

Combining the above with irreducibility we see that the subspace $\mathcal{N}_{n,n}$ is generated by the action of the group on the single element $N_{n,n}$.

Because the map (29) is surjective, the orthogonal complement of $\mathcal{N}_{n,n} \subseteq \mathcal{M}_n$ is a representation isomorphic to $\mathcal{M}_{n-1}$, which in turn contains the irreducible representation $\mathcal{N}_{n-1,n-1} \subseteq \mathcal{M}_{n-1}$ in the kernel of the trace map $\mathrm{tr}_{n-1} : \mathcal{M}_{n-1} \to \mathcal{M}_{n-2}$. Then, using Schur's Lemma again, there must be a subrepresentation $\mathcal{N}_{n-1,n} \subseteq \mathcal{M}_n$ that is isomorphic to $\mathcal{N}_{n-1,n-1} \subseteq \mathcal{M}_{n-1}$, which proves isomorphism (28). Proceeding inductively, we obtain the full decomposition (26).

To conclude the proof of Lemma 7 we need to show that $N_{j,n} \in \mathcal{N}_{j,n}$. By noting that

$$\mathrm{tr}_n [N_{j,n}] \propto N_{j,n-1} \in \mathcal{N}_{j,n-1} \tag{33}$$

is non-zero when $j < n$, we can proceed inductively to arrive at

$$(\mathrm{tr}_{j+1} \cdots \mathrm{tr}_{n-1} \mathrm{tr}_n)[N_{j,n}] \propto N_{j,j} \in \mathcal{N}_{j,j} \ , \tag{34}$$

which is the case analyzed above (32). The isomorphisms (28) provided by Schur's Lemma conclude the proof. □

The form Ω_ψ in $\mathcal{M}_n^d$ and $\mathcal{N}_n^d$

In this section we introduce a simple choice for the linear form Ω_ψ of Definition 4, for the cases $\mathbb{C}\mathcal{F}_d \cong \mathcal{M}_n^d$ and $\mathbb{C}\mathcal{F}_d \cong \mathcal{N}_n^d$. As already mentioned, this form encodes the structure of the set of mixed states.

Lemma 8. The linear form $\Omega_\psi : \mathcal{M}_n^d \to \mathbb{C}$ defined by

$$\Omega_\psi(M) = \mathrm{tr}\big(|\psi\rangle\langle\psi|^{\otimes n} M \big) \ , \tag{35}$$

is invariant under all stabilizer unitaries $U \in \mathrm{SU}(d, \psi)$

$$\Omega_\psi(U^{\otimes n} M U^{\otimes n \dagger}) = \Omega_\psi(M) \ , \tag{36}$$

and has support in all irreps $\mathcal{N}_j^d \subseteq \mathcal{M}_n^d$.

Proof. It is straightforward to check that the form (35) satisfies (36). To see that (35) has support in each irrep $\mathcal{N}_{j,n} \subseteq \mathcal{M}_n$, we observe that, for each j, there is a pure state ψ such that

$$\mathrm{tr}\big[N_{j,n} |\psi\rangle\langle\psi|^{\otimes n}\big] \neq 0 \ , \tag{37}$$

where $N_{j,n}$ is defined in (27). □

As mentioned above, these two constrains fix Ω_ψ up to an irrelevant proportionality factor in each irrep. To obtain Ω_ψ in the case $\mathbb{C}\mathcal{F}_d = \mathcal{N}_n^d$ we proceed in the following maner. Since $\mathcal{N}_n^d$ is a subrepresentation of $\mathcal{M}_n^d$ we can take (35) and perform the orthogonal projection onto the subspace $\mathcal{N}_{n,n}^d \subseteq \mathcal{M}_n^d$, defined via (27) or via the kernel fo the map (29).

Supplementary note 2: Multipartite systems

In this section we describe and impose the consistency constraints associated to composite systems and the star product.

Closedness under system composition

We require that any family of OPF sets $\mathcal{F}_2, \mathcal{F}_3, \ldots$ and $\mathcal{F}_\infty$ must be closed under system composition. This means that the complete set of measurements $\mathcal{F}_a$ of a system $\mathbb{C}^a$ also includes the measurements that appear in the description of $\mathbb{C}^a$ as part of a larger system $\mathbb{C}^a \otimes \mathbb{C}^b$.

Definition 9 (Closedness under system composition). If $\mathcal{F}_{ab}$ is the OPF set of $\mathbb{C}^a \otimes \mathbb{C}^b$ then the OPF set $\mathcal{F}_a$ of $\mathbb{C}^a$ is the following collection of functions

$$\mathrm{P}\mathbb{C}^a \to [0,1] \tag{38}$$

$$\alpha \mapsto \mathbf{f}(\alpha \otimes \beta) \tag{39}$$

for all $\mathbf{f} \in \mathcal{F}_{ab}$ and a fixed $\beta \in \mathrm{P}\mathbb{C}^b$, and all $a, b \in \{2, 3, \ldots, \infty\}$.

Note that the closure of $\mathcal{F}_{ab}$ under $\mathbb{1} \otimes \mathrm{SU}(b)$ implies that the set $\mathcal{F}_a$ defined via (38-39) does not depend on the choice of β. Also, it is straightforward to check that the OPF set $\mathcal{F}_a$ so defined satisfies all the requirements of Definition 1.

$\mathbb{C}\mathcal{F}_d \cong \mathcal{M}_n^d$

In the finite-dimensional case, closedness under system composition (Definition 9) implies the following strong fact. For any set of measurements $\mathcal{F}_{ab}$ of a bipartite system $\mathbb{C}^a \otimes \mathbb{C}^b$, the measurement spaces of the subsystems are $\mathbb{C}\mathcal{F}_a \cong \mathcal{M}_n^a$ for $\mathbb{C}^a$ and $\mathbb{C}\mathcal{F}_b \cong \mathcal{M}_n^b$ for $\mathbb{C}^b$, with the same n. In addition, using the fact that any pair of systems can be jointly described as a bipartite system, we conclude that all finite-dimensional systems $\mathbb{C}^d$ must have OPF space $\mathcal{M}_n^d$ (with the same value for n).

Lemma 10. For any pair of positive integers a, b, let $\mathcal{F}_{ab}$ be the OPF set of $\mathbb{C}^a \otimes \mathbb{C}^b$ with decomposition (Lemma 5)

$$\mathbb{C}\mathcal{F}_{ab} \cong \bigoplus_{j \in \mathcal{J}} \mathcal{N}_j^{ab} \ . \tag{40}$$

Define $\mathcal{F}_a$ as the set of functions

$$\mathrm{P}\mathbb{C}^a \to [0,1] \tag{41}$$

$$\alpha \mapsto \mathbf{f}(\alpha \otimes \beta) \tag{42}$$

for all $\mathbf{f} \in \mathcal{F}_{ab}$ and a fixed $\beta \in \mathrm{P}\mathbb{C}^b$. Then we have the $\mathrm{SU}(a)$-representation isomorphism

$$\mathbb{C}\mathcal{F}_a \cong \mathcal{M}_n^a \ , \tag{43}$$

where $n = \max \mathcal{J}$.

Note that, if we define $\mathcal{F}_b$ by exchanging the role of the subsystems $\mathbb{C}^a \otimes \mathbb{C}^b$ in (42), then we obtain the $\mathrm{SU}(b)$-representation isomorphism $\mathbb{C}\mathcal{F}_b \cong \mathcal{M}_n^b$ with the same value for n as in (43). Using the fact that any pair of systems can be jointly described as a bipartite system, we arrive at the following.

Corollary 11. Closedness under system composition (Definition 9) implies that all finite-dimensional Hilbert spaces $\mathbb{C}^d$ have OPF space $\mathbb{C}\mathcal{F}_d \cong \mathcal{M}_n^d$ with the same n.

Proof of Lemma 10. In order to establish the isomorphism (43) we analyze how the functions (42) transform under the subgroup $\mathrm{SU}(a) \otimes \mathbb{1}$. First, we do this in the case where $\mathcal{J}$ has finite cardinality, so that the decomposition (17) of $\mathbb{C}\mathcal{F}_{ab}$ has a largest irrep $\mathcal{N}_n^{ab}$. We further split this analysis into the case where the function $\mathbf{f}$ in (42) belongs to the subspace $\mathbf{f} \in \mathcal{N}_n^{ab} \subseteq \mathbb{C}\mathcal{F}_{ab}$, and the general case.

Using the characterization of $\mathcal{N}_n^{ab}$ as the kernel of the map (29) we can say the following. For each $\mathbf{f} \in \mathcal{N}_n^{ab} \subseteq \mathbb{C}\mathcal{F}_{ab}$ there is a matrix $F \in \mathcal{M}_n^{ab}$ such that $\mathrm{tr}_n F = 0$ and

$$\mathbf{f}(\alpha \otimes \beta) = \mathrm{tr}\big(F\, |\alpha\rangle\langle\alpha|^{\otimes n} \otimes |\beta\rangle\langle\beta|^{\otimes n}\big) \ , \tag{44}$$

for all α, β. (Note that, in order to improve clarity, we re-arranged the order of the tensor factors.) The matrices

$$|\alpha\rangle\langle\alpha|^{\otimes n} \otimes |\beta\rangle\langle\beta|^{\otimes n} \in \mathcal{M}_n^{ab} \ , \tag{45}$$

are contained in the subspace

$$\begin{aligned} |\alpha\rangle\langle\alpha|^{\otimes n} \otimes |\beta\rangle\langle\beta|^{\otimes n} &\in P_+^{\mathsf{A}} P_+^{\mathsf{B}} \mathcal{M}_n^{ab} P_+^{\mathsf{A}} P_+^{\mathsf{B}} \\ &\cong \mathcal{M}_n^{a} \otimes \mathcal{M}_n^{b} \\ &\cong \bigoplus_{j,j'=0}^{n} \mathcal{N}_j^{a} \otimes \mathcal{N}_{j'}^{b} \ , \end{aligned} \tag{46}$$

where the isomorphisms are of $\mathrm{SU}(a) \otimes \mathrm{SU}(b)$ representations. Even more, using the full support conditions (37) in each tensor factor, we conclude that the matrices $|\alpha\rangle\langle\alpha|^{\otimes n} \otimes |\beta\rangle\langle\beta|^{\otimes n}$ generate the whole space (46).

Next we analyze the $\mathrm{SU}(a) \otimes \mathrm{SU}(b)$ action on the function (44), which is the action on the intersection between the subspaces $\{F \in \mathcal{M}_n^{ab} : \mathrm{tr}_n F = 0\}$ and (46). This intersection can be characterized by writing the trace as

$$\mathrm{tr}_n = \mathrm{tr}_{\mathsf{A}_n} \mathrm{tr}_{\mathsf{B}_n} \ , \tag{47}$$

where $\mathrm{tr}_{\mathsf{A}_n}$ is the trace on the nth factor of $\mathcal{M}_n^a$, and $\mathrm{tr}_{\mathsf{B}_n}$ is the trace on the nth factor of $\mathcal{M}_n^b$. The above identity implies that if $\mathrm{tr}_{\mathsf{A}_n} F = 0$ or $\mathrm{tr}_{\mathsf{B}_n} F = 0$ then $\mathrm{tr}_n F = 0$. Therefore, the above-mentioned intersection contains all irreps $\mathcal{N}_n^a \otimes \mathcal{N}_j^b$ and $\mathcal{N}_j^a \otimes \mathcal{N}_n^b$ for $j = 0, 1, \ldots, n$. This implies that the $\mathrm{SU}(a) \otimes \mathbb{1}$ action on the space of functions (44) with $\mathbf{f} \in \mathcal{N}_n^{ab} \subseteq \mathbb{C}\mathcal{F}_{ab}$ decomposes into the irreps $\mathcal{N}_0^a, \ldots, \mathcal{N}_n^a$, with possible repetitions.

In the general case $\mathbf{f} \in \mathbb{C}\mathcal{F}_{ab}$, the addition of all subspaces $\mathcal{N}_j^{ab} \subseteq \mathcal{F}_{ab}$ with $j < n$ does not add any new irrep to the list $\mathcal{N}_0^a, \ldots, \mathcal{N}_n^a$. Although it may increase the repetitions.

Finally, we establish the desired isomorphism (43) by recalling Lemma 5. This tells us that any OPF set, like the $\mathcal{F}_a$ defined through (42), has no repeated irreps. □

The star product

In this subsection we introduce the star product, which contains the information of which measurements of a composite system $\mathcal{F}_{ab}$ are local.

Definition 12. The star product is a map $\star : \mathcal{F}_a \times \mathcal{F}_b \to \mathcal{F}_{ab}$ defined on any pair of OPF sets $\mathcal{F}_a, \mathcal{F}_b$, with the following properties:

- preserves the local structure
$$(\mathbf{f} \star \mathbf{u})(\alpha \otimes \beta) = \mathbf{f}(\alpha) \ , \tag{48}$$
- preserves probability
$$\mathbf{u}_{\mathsf{A}} \star \mathbf{u}_{\mathsf{B}} = \mathbf{u}_{\mathsf{AB}} \ , \tag{49}$$
$$\mathbf{f}_{\mathsf{A}} \star \mathbf{0}_{\mathsf{B}} = \mathbf{0}_{\mathsf{AB}} \tag{50}$$
- commutes with local mixing operations
$$\left(\textstyle\sum_x p_x\, \mathbf{f}^x\right) \star \mathbf{g} = \textstyle\sum_x p_x \left(\mathbf{f}^x \star \mathbf{g}\right) \ , \tag{51}$$
- commutes with the local group action
$$(\mathbf{f} \circ U) \star \mathbf{g} = (\mathbf{f} \star \mathbf{g}) \circ (U \otimes \mathbb{1}) \ , \tag{52}$$
- and it is associative
$$(\mathbf{f} \star \mathbf{g}) \star \mathbf{h} = \mathbf{f} \star (\mathbf{g} \star \mathbf{h}) \ , \tag{53}$$

for any $\mathbf{f}, \mathbf{f}^x \in \mathcal{F}_a$; $\mathbf{g} \in \mathcal{F}_b$; $\mathbf{h} \in \mathcal{F}_c$; $\alpha \in \mathrm{P}\mathbb{C}^a$; $\beta \in \mathrm{P}\mathbb{C}^b$; $U \in \mathrm{U}(a)$; $a, b \in \{2, 3, \ldots, \infty\}$, and any probability distribution p_x. Properties (48-53) must also hold when exchanging factors.

The $\star$-product allows us to write the reduced state of a bipartite pure state $\psi \in \mathbb{C}^a \otimes \mathbb{C}^b$ on the subsystem $\mathbb{C}^a$ as the linear form $\mathbf{f} \mapsto \Omega_\psi(\mathbf{f} \star \mathbf{u})$ for all $\mathbf{f} \in \mathbb{C}\mathcal{F}_a$.

Note that property (48) is weaker than the analog condition in the main text:

$$(\mathbf{f} \star \mathbf{g})(\alpha \otimes \beta) = \mathbf{f}(\alpha)\mathbf{g}(\beta) \ . \tag{54}$$

The reason for writing the stronger condition in the main text is that it does not require $\mathbf{u}$ to be defined. The following lemma proves that, in our context, condition (48) implies condition (54).

Lemma 13. Suppose that any ensemble (ψ_r, p_r) satisfying

$$\sum_r p_r \, \mathbf{f}(\psi_r) = \mathbf{f}(\varphi) \ , \quad \forall \mathbf{f} \in \mathcal{F}_d \ , \tag{55}$$

is of the form $\psi_r = \varphi$ for all r. Then (48) implies

$$(\mathbf{f} \star \mathbf{g})(\alpha \otimes \beta) = \mathbf{f}(\alpha) \, \mathbf{g}(\beta) \ . \tag{56}$$

Proof. First, let $\{\mathbf{g}_i\}$ be a complete measurement and define the following probabilities and the (not necessarily pure) states

$$p_i = \mathbf{g}_i(\beta) \ , \tag{57}$$

$$\Omega_i(\mathbf{f}) = \begin{cases} (\mathbf{f} \star \mathbf{g}_i)(\alpha \otimes \beta)/p_i & \text{if } p_i \neq 0 \\ \mathbf{f}(\alpha) & \text{if } p_i = 0 \end{cases} \ , \tag{58}$$

for all $\mathbf{f}$. Second, substitute $\sum_i \mathbf{g}_i = \mathbf{u}$ in (48) obtaining

$$\sum_i p_i \, \Omega_i(\mathbf{f}) = \mathbf{f}(\alpha) \ , \tag{59}$$

for all $\mathbf{f}$. Third, use the premise of the lemma to conclude that

$$\Omega_i(\mathbf{f}) = \mathbf{f}(\alpha) \tag{60}$$

for all i and $\mathbf{f}$. Finally, substituting back the definition of Ω_i we obtain

$$(\mathbf{f} \star \mathbf{g}_i)(\alpha \otimes \beta) = \mathbf{f}(\alpha) \, \mathbf{g}_i(\beta) \ , \tag{61}$$

which implies (56). □

By "preservation of probability" (49) it is meant that the fact that all outcome probabilities add up to one

$$\sum_i \mathbf{f}_i(\alpha) = 1 \ , \tag{62}$$

is independent of whether we describe a system on its own or as part of a larger system

$$\sum_i (\mathbf{f}_i \star \mathbf{u}_\mathsf{B})(\psi_\mathsf{AB}) = 1 \ . \tag{63}$$

Also, the joint outcome $\mathbf{f}_\mathsf{A} \star \mathbf{0}_\mathsf{B}$, where $\mathbf{0}_\mathsf{B}$ is the formal outcome with zero probability for all states, must have zero probability, which gives (50).

The action of the $*$-product is not defined on the elements of $\mathbb{C}\mathcal{F}_a$ that are not in $\mathcal{F}_a$. However, the following lemma shows that one can define the action of the $*$-product to the rest of elements of $\mathbb{C}\mathcal{F}_a$ in such a way that the map is bilinear.

Lemma 14. Any star-product map $\star : \mathcal{F}_a \times \mathcal{F}_b \to \mathcal{F}_{ab}$ as specified in Definition 12 can be extended to a bilinear map $\star : \mathbb{C}\mathcal{F}_a \times \mathbb{C}\mathcal{F}_b \to \mathbb{C}\mathcal{F}_{ab}$ with the same properties (48-53).

Proof. For any given $\mathbf{g} \in \mathcal{F}_b$ define the map

$$\xi : \mathcal{F}_a \to \mathcal{F}_{ab} \ , \tag{64}$$

$$\mathbf{f} \mapsto \mathbf{f} \star \mathbf{g} \ . \tag{65}$$

Using Definition 12 we obtain the following properties for the map

$$\xi(\mathbf{0}) = \mathbf{0} \ , \tag{66}$$

$$\xi(\textstyle\sum_x p_x \mathbf{f}^x) = \sum_x p_x \, \xi(\mathbf{f}^x) \ , \tag{67}$$

for any probability distribution p_x. In Appendix 1 of [3] it is proven that it is possible to define a $\mathbb{R}$-linear map $\xi' : \mathbb{R}\mathcal{F}_a \to \mathbb{R}\mathcal{F}_{ab}$ which is identical to ξ inside $\mathcal{F}_a$. Finally, we can define the $\mathbb{C}$-linear map $\xi'' : \mathbb{C}\mathcal{F}_a \to \mathbb{C}\mathcal{F}_{ab}$ in the natural way

$$\xi''(\mathbf{f}_1 + \mathrm{i}\mathbf{f}_2) = \xi'(\mathbf{f}_1) + \mathrm{i}\, \xi'(\mathbf{f}_2) \tag{68}$$

for any pair $\mathbf{f}_1, \mathbf{f}_2 \in \mathbb{R}\mathcal{F}_a$.

The above construction can be repeated with an exchange of parties. Proving the desired result. □

Lemma 15. In the case $\mathbb{C}\mathcal{F}_d \cong \mathcal{M}_n^d$ we have the identity

$$P_+^{\mathsf{A}} P_+^{\mathsf{B}} \left(\mathcal{M}_n^a \star \mathcal{M}_n^b\right) P_+^{\mathsf{A}} P_+^{\mathsf{B}} = \mathcal{M}_n^a \otimes \mathcal{M}_n^b \ , \tag{69}$$

of $\mathrm{SU}(a) \otimes \mathrm{SU}(b)$ representations.

Proof. If we write condition (56) with the form Ω_α introduced in (35) then we get

$$\begin{aligned} &\mathrm{tr}\big([F \star G] |\alpha\rangle\langle\alpha|^{\otimes n} \otimes |\beta\rangle\langle\beta|^{\otimes n}\big) \\ &\quad = \mathrm{tr}\big(F|\alpha\rangle\langle\alpha|^{\otimes n}\big)\, \mathrm{tr}\big(G|\beta\rangle\langle\beta|^{\otimes n}\big) \\ &\quad = \mathrm{tr}\big([F \otimes G] |\alpha\rangle\langle\alpha|^{\otimes n} \otimes |\beta\rangle\langle\beta|^{\otimes n}\big) \ , \end{aligned} \tag{70}$$

for all $F \in \mathcal{M}_n^a$, $G \in \mathcal{M}_n^b$, $\alpha \in \mathrm{P}\mathbb{C}^a$ and $\beta \in \mathrm{P}\mathbb{C}^b$. By noting that the set of matrices $|\alpha\rangle\langle\alpha|^{\otimes n} \otimes |\beta\rangle\langle\beta|^{\otimes n}$ span the subspace $P_+^{\mathsf{A}} P_+^{\mathsf{B}} \mathcal{M}_n^{ab} P_+^{\mathsf{A}} P_+^{\mathsf{B}} \subseteq \mathcal{M}_n^{ab}$ we can write (70) as

$$P_+^{\mathsf{A}} P_+^{\mathsf{B}} \left(F \star G\right) P_+^{\mathsf{A}} P_+^{\mathsf{B}} = F \otimes G \ , \tag{71}$$

for all F, G. This proves identity (69). □

Supplementary note 3: Non-associativity of $\star : \mathcal{M}_a \times \mathcal{M}_b \to \mathcal{M}_{ab}$

The permutation group and Schur-Weyl duality

In this section we review some well-known results of representation theory. The n-th tensor-power of a vector space $\mathbb{C}^d$ can be decomposed as

$$\left(\mathbb{C}^d\right)^{\otimes n} \cong \bigoplus_\lambda \mathcal{V}_\lambda^d \otimes \mathcal{S}_\lambda^n \ , \tag{72}$$

where λ runs over all partitions of n with at most d parts, $\mathcal{V}_\lambda^d$ are irreps of $\mathrm{SU}(d)$, and $\mathcal{S}_\lambda^n$ are the irreps of the group of permutations of n objects. The partition $\lambda = (n)$ corresponds to the trivial representation of the group of permutations, and hence, all vectors in the subspace $\mathcal{V}_{(n)}^d \otimes \mathcal{S}_{(n)}^n$ are permutation-invariant. Because of this, this subspace and the corresponding projector $P_+ = P_{(n)}$ are called symmetric.

When considering a bipartite space $\mathbb{C}^d = \mathbb{C}^a \otimes \mathbb{C}^b$, the symmetric projector can be written as

$$P_+^{\mathsf{AB}} = \sum_\lambda Q_\lambda^{\mathsf{AB}} \ , \tag{73}$$

where Q_λ^{AB} is the orthogonal projector onto the subspace of $[\mathcal{V}_\lambda^a \otimes \mathcal{S}_\lambda^n]^{\mathsf{A}} \otimes [\mathcal{V}_\lambda^b \otimes \mathcal{S}_\lambda^n]^{\mathsf{B}}$ that transforms trivially when applying the same permutation to A and B. Specifically, we can write it as

$$Q_\lambda^{\mathsf{AB}} = \mathbb{1}_{\mathcal{V}_\lambda^a} \otimes \mathbb{1}_{\mathcal{V}_\lambda^b} \otimes |\tau\rangle_{\mathcal{S}_\lambda^n \mathcal{S}_\lambda^n} \langle\tau| \tag{74}$$

where $\mathbb{1}_{\mathcal{V}}$ is the identity on the subspace $\mathcal{V}$ and

$$|\tau_\lambda\rangle_{\mathcal{V}\mathcal{V}'} = \sum_k |k\rangle_{\mathcal{V}} \otimes |k\rangle_{\mathcal{V}'} \tag{75}$$

is the "maximally entangled state" of the product space $\mathcal{V} \otimes \mathcal{V}'$. The invariance of $|\tau_\lambda\rangle_{\mathsf{AB}}$ under identical permutations on A and B is analogous to the invariance of any maximally entangled state under transformations of the form $U \otimes U^*$, together with the fact that all irreps of the permutation group are real (self-dual).

In the tri-partite case $\mathbb{C}^d = \mathbb{C}^a \otimes \mathbb{C}^b \otimes \mathbb{C}^c$, the symmetric projector can be written as

$$P_+^{\mathsf{ABC}} = \sum_{\lambda,\mu,\nu} Q_{\lambda,\mu,\nu}^{\mathsf{ABC}} \ , \tag{76}$$

where $Q_{\lambda,\mu,\nu}^{\mathsf{ABC}}$ is the orthogonal projector onto the subspace of $[\mathcal{V}_\lambda^a \otimes \mathcal{S}_\lambda^n]^{\mathsf{A}} \otimes [\mathcal{V}_\mu^b \otimes \mathcal{S}_\mu^n]^{\mathsf{B}} \otimes [\mathcal{V}_\nu^c \otimes \mathcal{S}_\nu^n]^{\mathsf{C}}$ that transforms trivially when applying the same permutation on $\mathsf{A}, \mathsf{B}, \mathsf{C}$. Therefore, the projector $Q_{\lambda,\mu,\nu}^{\mathsf{ABC}}$ is zero unless the irrep decomposition of $\mathcal{S}_\lambda^n \otimes \mathcal{S}_\mu^n \otimes \mathcal{S}_\nu^n$ contains the trivial $\mathcal{S}_{(n)}^n$. Particularly, when one of the three partitions is (n), we recover the bipartite case

$$Q_{(n),\lambda,\lambda}^{\mathsf{ABC}} = \mathbb{1}_{\mathcal{V}_{(n)}^a} \otimes \mathbb{1}_{\mathcal{V}_\lambda^b} \otimes \mathbb{1}_{\mathcal{V}_\lambda^c} \otimes |\tau\rangle_{\mathcal{S}_\lambda^n \mathcal{S}_\lambda^n}^{\mathsf{BC}} \langle\tau| \ , \tag{77}$$

for all λ. That is, if one partition is (n) then the other two partitions have to be equal. And this is why, in the bipartite case, the projector Q_λ^{AB} only depends on one partition.

The irreducible representations of $\mathrm{SU}(d)$

Using the Littlewood-Richardson rule [2], we can decompose $\mathcal{V}_\lambda^d \otimes \mathcal{V}_\mu^d$ into irreps, and prove the following patterns.

Lemma 16. The decomposition of $\mathcal{V}_{(n-1,1)}^4 \otimes \mathcal{V}_{(n-1,1)}^{4*}$ into irreps does not include any $\mathcal{N}_j^4$ with $j \geq n$.

Proof. If we denote by λ^* the partition of the irrep $\mathcal{V}_{(n-1,1)}^{4*}$, and by λ_j the partition of $\mathcal{N}_j^4$, then we have

$$\lambda^* = (n-1, n-1, n-2) \ , \tag{78}$$
$$\lambda_j = (2j, j, j) \ , \tag{79}$$
$$\lambda = (n-1, 1) \ . \tag{80}$$

Applying the Littlewood-Richardson rule [2] to the Young tableaux λ and λ^*, we see that all resulting tableaux have at most $4(n-1)$ boxes, while the tableau of λ_j has $4j$ boxes. Therefore, no tableau λ_j with $j \geq n$ can appear in the product of λ and λ^*. □

Lemma 17. When restricting the irrep $\mathcal{N}_n^4$ of SU(4) to any SU(2) subgroup, the decomposition of $\mathcal{N}_n^4$ into irreps of SU(2) does not include any $\mathcal{N}_j^2$ with $j > n$.

Proof. The SU(2) irreps in $\mathcal{N}_n^4$ correspond to straight lines of weights in the weight diagram of $\mathcal{N}_n^4$. The longest such line contains $2n+1$ weights. Therefore, the largest SU(2) irrep is $\mathcal{N}_n^2$. □

Proof of the main theorem

The following theorem shows that only in the quantum cas (that is $n = 1$) there is an associative star product $\star : \mathcal{M}_n^a \times \mathcal{M}_n^b \to \mathcal{M}_n^{ab}$.

Theorem 18. If $n \geq 2$ then there is no bilinear map $\star : \mathcal{M}_n^a \times \mathcal{M}_n^b \to \mathcal{M}_n^{ab}$ satisfying the star-product Definition 12.

Proof. Analysis of bipartite systems. Let us consider a bipartite system with Hilbert space $\mathbb{C}^a \otimes \mathbb{C}^b$ and dimensions $a = 2$ and $b = 4$. Using the decomposition (73) of the projector P_+^{AB} onto the symmetric subspace of $(\mathbb{C}^a \otimes \mathbb{C}^b)^{\otimes n}$ we can decompose $\mathcal{M}_n^{ab}$ into subspaces as

$$\mathcal{M}_n^{ab} = \sum_{\lambda,\mu} Q_\lambda^{\mathsf{AB}} \mathcal{M}_n^{ab} Q_\mu^{\mathsf{AB}} \ , \tag{81}$$

each labeled by a pair of n-partitions (λ, μ). Since system A is 2-dimensional all n-partitions λ, μ have at most two rows. The action of $\mathrm{SU}(ab)$ might not be well-defined in some of these subspaces, but the action of the subgroup $\mathrm{SU}(a) \otimes \mathrm{SU}(b) \subseteq \mathrm{SU}(ab)$ is well-defined in each (λ, μ) subspace from (81). Concretely, we have the following isomorphism of $\mathrm{SU}(a) \otimes \mathrm{SU}(b)$ representations

$$Q_\lambda^{\mathsf{AB}} \mathcal{M}_n^{ab} Q_\mu^{\mathsf{AB}} \cong \mathcal{V}_\lambda^a \otimes \mathcal{V}_\mu^{a*} \otimes \mathcal{V}_\lambda^b \otimes \mathcal{V}_\mu^{b*} \ , \tag{82}$$

which in particular gives

$$Q_+^{\mathsf{AB}} \mathcal{M}_n^{ab} Q_+^{\mathsf{AB}} \cong \mathcal{M}_n^a \otimes \mathcal{M}_n^b \ . \tag{83}$$

Now, let us take the subspace (82) corresponding to $\lambda = \mu = (n-1,1)$, and decompose it into two orthogonal subspaces

$$Q^{\mathsf{AB}}_{(n-1,1)} \mathcal{M}^{ab}_n Q^{\mathsf{AB}}_{(n-1,1)} = \mathcal{W}^b_{\text{licit}} \oplus \mathcal{W}^b_{\text{illicit}} \ , \tag{84}$$

defined in the following way: (i) consider the action of the subgroup $\mathbb{1} \otimes \mathrm{SU}(b)$ on the left-hand side of (84), (ii) decompose this action into irreps of $\mathrm{SU}(b)$, (iii) let $\mathcal{W}^b_{\text{licit}}$ be the direct sum of all irreps $\mathcal{N}^b_j$ for any j, and (iv) let $\mathcal{W}^b_{\text{illicit}}$ be the direct sum of the rest of irreps. The super-index b in these subspaces $\mathcal{W}^b_{\text{xxlicit}}$ reminds us that these are $\mathrm{SU}(b)$ representations.

Lemma 16 tells us that $\mathcal{W}^b_{\text{licit}}$ does not contain any $\mathcal{N}^b_j$ with $j \geq n$. That is

$$\mathcal{W}^b_{\text{licit}} \cong \bigoplus_{j<n} \mathcal{N}^b_j \ , \tag{85}$$

where the sum over j may contain some absences and repetitions. Combining this with Schur's Lemma and the commutativity constraint (52), we see that the image of the $\star$-product $[\mathcal{M}^a_n \star \mathcal{M}^b_n] \subseteq \mathcal{M}^{ab}_n$ does not have support in $\mathcal{W}^b_{\text{illicit}}$. In particular,

$$Q^{\mathsf{AB}}_{(n-1,1)} \left[\mathbf{u}_{\mathsf{A}} \star \mathcal{M}^b_n\right] Q^{\mathsf{AB}}_{(n-1,1)} \subseteq \mathcal{W}^b_{\text{licit}} \ . \tag{86}$$

In the next subsection we show that, if B is itself considered a bipartite system then the above subspace contains the irrep $\mathcal{N}^b_n$, which is incompatible with (85) and (86).

Analysis of tripartite systems. Now let us describe system B as a bipartite system CE with Hilbert space $\mathbb{C}^b = \mathbb{C}^c \otimes \mathbb{C}^e$ and dimensions $c = e = 2$. Combining the decompositions of the bipartite (73) and the tripartite (76) symmetric projectors, we can write

$$Q^{\mathsf{AB}}_{(n-1,1)} = \sum_{\mu,\nu} Q^{\mathsf{ACE}}_{(n-1,1),\mu,\nu} \ . \tag{87}$$

Now, if we substitute the decomposition (87) into (86) and remove all terms except for the $\mu = (n)$ and $\nu = (n-1,1)$ one, the inclusion still holds

$$Q^{\mathsf{ACE}}_{(n-1,1),(n),(n-1,1)} \left[\mathbf{u}_{\mathsf{A}} \star \mathcal{M}^{ce}_n\right] Q^{\mathsf{ACE}}_{(n-1,1),(n),(n-1,1)} \subseteq \mathcal{W}^{ce}_{\text{licit}}. \tag{88}$$

Importantly, the projector $Q^{\mathsf{ACE}}_{(n-1,1),(n),(n-1,1)}$ is non-zero according to (77).

Now, if we restrict the action of $\mathbb{1} \otimes \mathrm{SU}(ce)$ to the subgroup $\mathbb{1} \otimes \mathrm{SU}(c) \otimes \mathbb{1}$ and use (85) and Lemma 17, then we see that the irrep decompositions of the right-hand sides of (88) does not contain $\mathcal{N}^c_n$. Also, due to the fact that the subspace

$$Q^{\mathsf{ACE}}_{(n-1,1),(n),(n-1,1)} \left[\mathbf{u}_{\mathsf{A}} \star \mathcal{M}^c_n \star \mathbf{u}_{\mathsf{E}}\right] Q^{\mathsf{ACE}}_{(n-1,1),(n),(n-1,1)} \tag{89}$$

is a subrepresentation of (88), it does not contain the irrep $\mathcal{N}^c_n$. Next we show that this is incompatible with associativity. Using Lemma 15 and recalling that

$$\mathbf{u}_{\mathsf{CE}} = P^{\mathsf{CE}}_+ = \sum_\lambda Q^{\mathsf{CE}}_\lambda \tag{90}$$

we obtain the isomorphism

$$Q^{\mathsf{ACE}}_{(n),(n-1,1),(n-1,1)} \left[\mathcal{M}^a_n \star \mathbf{u}_{\mathsf{CE}}\right] Q^{\mathsf{ACE}}_{(n),(n-1,1),(n-1,1)} \cong \mathcal{M}^a_n$$

of $\mathrm{SU}(a) \otimes \mathbb{1} \otimes \mathbb{1}$ representations, which include the irrep $\mathcal{N}^a_n$. By permuting the subsystems ACE we conclude that the $\mathbb{1} \otimes \mathrm{SU}(c) \otimes \mathbb{1}$ representation (89) also contains the irrep $\mathcal{N}^c_n$, in contradiction with our previous conclusion! □

At this point we can contrast the above argument with the disregarded case $n = 1$. In this cse there is only one partition $\lambda = \mu = (1)$, and

$$Q^{\mathsf{AB}}_\lambda \mathcal{M}^{ab}_1 Q^{\mathsf{AB}}_\mu = \mathcal{M}^{ab}_1 = \mathcal{M}^a_1 \otimes \mathcal{M}^b_1 = \mathcal{W}^b_{\text{licit}} \ ,$$

which implies that $\mathcal{W}^b_{\text{illicit}}$ is trivial. Therefore the above contradiction does not apply to the $n = 1$ case.

Corollary 19 (measurement theorem). Any family of OPF sets $\mathcal{F}_d$ with finite d, equipped with a $\star$-product, and satisfying the assumptions "possibility of state estimation" and "closedness under system composition", has OPFs and $\star$-product of the form

$$\mathbf{f}(\varphi) = \langle\varphi|F|\varphi\rangle \ , \tag{91}$$

$$(\mathbf{f} \star \mathbf{g})(\psi) = \langle\psi|F \otimes G|\psi\rangle \ , \tag{92}$$

for all normalized $\varphi \in \mathbb{C}^a$ and $\psi \in \mathbb{C}^a \otimes \mathbb{C}^b$, where the $\mathbb{C}^a$-matrix F satisfies $0 \leq F \leq \mathbb{1}$, and analogously for G.

Supplementary note 4: Countably infinite-dimensional Hilbert spaces

Since all countably infinite-dimensional Hilbert spaces are isomorphic, we denote them all by $\mathbb{C}^\infty$. The topological space of all one-dimensional subspaces of $\mathbb{C}^\infty$ is denoted by $\mathrm{P}\mathbb{C}^\infty$. Also, for any given subspace $S \subseteq \mathbb{C}^\infty$ we denote the corresponding orthogonal projector by Π_S.

The following lemma tells us that the measurements on $\mathbb{C}^\infty$ are of the quantum form (91) if and only if they have such form when restricted to any finite-dimensional subspaces of $\mathbb{C}^\infty$.

Lemma 20. For every (not necessarily continuous) function $\mathbf{f} : \mathrm{P}\mathbb{C}^\infty \to [0,1]$, the following two statements are equivalent:

- There exists a self-adjoint operator F such that $0 \leq F \leq \mathbb{1}$ and $\mathbf{f}(\psi) = \langle\psi|F|\psi\rangle$ for all normalized $\psi \in \mathbb{C}^\infty$.
- For every finite-dimensional subspace $S \subset \mathbb{C}^\infty$, there exists a self-adjoint operator F_S fully supported on S, i.e. $\Pi_S F_S \Pi_S = F_S$, such that $0 \leq F_S \leq \mathbb{1}$ and $\mathbf{f}(\psi) = \langle\psi|F_S|\psi\rangle$ for all normalized $\psi \in S$.

Proof. Suppose the first statement, $\mathbf{f}(\psi) = \langle\psi|F|\psi\rangle$. Then, for every finite-dimensional subspace S, define $F_S = \Pi_S F \Pi_S$. Now, it is clear that for all normalized $\psi \in S$, we have

$$\langle\psi|F_S|\psi\rangle = \langle\psi|F|\psi\rangle = \mathbf{f}(\psi) \tag{93}$$

which is the second statement of the lemma.

Conversely, suppose that for every finite-dimensional subspace $S \subset \mathbb{C}^\infty$ there exists F_S satisfying $\Pi_S F_S \Pi_S = F_S$ and $\mathbf{f}(\psi) = \langle\psi|F_S|\psi\rangle$ for all normalized $\psi \in S$. First we prove the following intermediate claim: *Let* $(S^{(n)})_{n\in\mathbb{N}}$ *be any sequence of subspaces with* $\dim S^{(n)} = n$ *and* $S^{(n)} \subset S^{(n+1)}$ *such that for* $S := \bigcup_{n\in\mathbb{N}} S^{(n)}$ *we get the norm closure* $\bar{S} = \mathbb{C}^\infty$. *Then there exists a unique bounded operator* F *on* $\mathbb{C}^\infty$ *such that* $\mathbf{f}(\psi) = \langle\psi|F|\psi\rangle$ *for all normalized states* $\psi \in S$.

To prove this, note that the sequence of subspaces defines a unique orthonormal basis $\{|i\rangle\}_{i\in\mathbb{N}}$ of $\mathbb{C}^\infty$ such that $S^{(n)} = \mathrm{span}\{|1\rangle, |2\rangle, \ldots, |n\rangle\}$ (this follows e.g. from Gram-Schmidt orthogonalization). Define the projector $\Pi^{(n)} = \sum_{i=1}^n |i\rangle\langle i|$ onto $S^{(n)}$, and define the self-adjoint operator $F^{(n)} = F_{S^{(n)}}$ whose existence we have assumed as a premise. It satisfies $\mathbf{f}(\psi) = \langle\psi|F^{(n)}|\psi\rangle$ for all normalized $\psi \in S^{(n)}$ and $\Pi^{(n)} F^{(n)} \Pi^{(n)} = F^{(n)}$ as well as $0 \leq F^{(n)} \leq \mathbb{1}$.

Now, fix any vector $\psi \in \mathbb{C}^\infty$ and define the family $\varphi^{(n)} = F^{(n)}\psi \in S^{(n)}$. Let $m \leq n$, and note that every $\alpha \in S^{(m)} \subset S^{(n)}$ satisfies $\langle\alpha|F^{(m)}|\alpha\rangle = \mathbf{f}(\alpha) = \langle\alpha|F^{(n)}|\alpha\rangle$, and thus, by polarization, we also have that $\langle\alpha|F^{(m)}|\beta\rangle = \langle\alpha|F^{(n)}|\beta\rangle$ for all $\alpha, \beta \in S^{(m)}$.

Now, define the sequences of complex numbers $x_j^{(n)} = \langle j|\varphi^{(n)}\rangle$ and $y_j = \langle j|\psi\rangle$. For any $j \leq m$ we have

$$\begin{aligned}\left|x_j^{(n)} - x_j^{(m)}\right|^2 &= \left|\langle j|\varphi^{(n)}\rangle - \langle j|\varphi^{(m)}\rangle\right|^2 = \left|\langle j|F^{(n)}|\psi\rangle - \langle j|F^{(m)}|\psi\rangle\right|^2 = \left|\langle j|F^{(n)}\textstyle\sum_{i=m+1}^n y_i|i\rangle\right|^2 \\ &\leq \left\|\textstyle\sum_{i=m+1}^n y_i|i\rangle\right\|^2 = \textstyle\sum_{i=m+1}^n |y_i|^2 \leq \displaystyle\sum_{i=m+1}^\infty |y_i|^2 \xrightarrow{m\to\infty} 0 \;.\end{aligned}$$

Hence, for every j, the sequence $(x_j^{(n)})_{n\in\mathbb{N}}$ is a Cauchy sequence, which has some limit $x_j = \lim_{n\to\infty} x_j^{(n)}$.

For all $N \in \mathbb{N}$ we have $\sum_{j=1}^N |x_j^{(n)}|^2 \leq \|\varphi^{(n)}\|^2 \leq \|\psi\|^2$, and thus $\sum_{j=1}^N |x_j|^2 = \lim_{n\to\infty} \sum_{j=1}^N |x_j^{(n)}|^2 \leq \|\psi\|^2$. This implies that the object $\varphi = \sum_{j=1}^\infty x_j|j\rangle$ has finite norm $\|\varphi\|^2 = \sum_{j=1}^\infty |x_j|^2 \leq \|\psi\|^2$, and it is therefore a vector $\varphi \in \mathbb{C}^\infty$.

The above construction produces one output vector $\varphi \in \mathbb{C}^\infty$ for each input vector $\psi \in \mathbb{C}^\infty$. This defines a map $F : \mathbb{C}^\infty \to \mathbb{C}^\infty$ via $F(\psi) = \varphi$. Moreover, it is easy to check that $F(\lambda\psi) = \lambda F(\psi)$ for any $\lambda \in \mathbb{C}$, and $F(\psi + \psi') = F(\psi) + F(\psi')$ for any $\psi, \psi' \in \mathbb{C}^\infty$. Hence F is a linear operator. Since $\|F(\psi)\| \leq \|\psi\|$ the operator F is bounded and hence continuous.

Suppose $\psi \in S$, then there exists some $n \in \mathbb{N}$ such that $\psi \in S^{(n)}$. By construction of F, for all $j \in \mathbb{N}$, we have

$$\langle j|F|\psi\rangle = \langle j|\varphi\rangle = x_j = \lim_{k\to\infty} x_j^{(k)} = \lim_{k\to\infty} \langle j|\varphi^{(k)}\rangle = \lim_{k\to\infty} \langle j|F^{(k)}|\psi\rangle.$$

In particular, if $1 \leq j \leq n$, then $|j\rangle, \psi \in S^{(n)}$, and so $\langle j|F^{(k)}|\psi\rangle = \langle j|F^{(n)}|\psi\rangle$ for all $k \geq n$, thus $\langle j|F|\psi\rangle = \langle j|F^{(n)}|\psi\rangle$. We thus obtain

$$\mathbf{f}(\psi) = \langle\psi|F^{(n)}|\psi\rangle = \sum_{j=1}^n \bar{y}_j \langle j|F^{(n)}|\psi\rangle = \sum_{j=1}^n \bar{y}_j \langle j|F|\psi\rangle = \langle\psi|F|\psi\rangle.$$

This proves *existence* in our intermediate claim, now we would like to prove *uniqueness*. To this end, suppose that both F and G are bounded operators such that $\mathbf{f}(\psi) = \langle\psi|F|\psi\rangle = \langle\psi|G|\psi\rangle$ for all normalized $\psi \in S$.

Then the bounded operator $\Delta := F - G$ satisfies $\langle\psi|\Delta|\psi\rangle = 0$ for all $\psi \in S$. Since every vector in $\mathbb{C}^\infty$ can be approximated in norm to arbitary accuracy by elements in S, and since Δ is continuous, this shows that $\langle\psi|\Delta|\psi\rangle = 0$ for all $\psi \in \mathbb{C}^\infty$, and thus $\Delta = 0$ since Δ is bounded and the Hilbert space is complex [4].

This proves our intermediate claim. Since $\mathbf{f}(\psi) \in [0,1]$ for all normalized $\psi \in S$, and all normalized vectors in $\mathbb{C}^\infty$ can be approximated in norm by normalized vectors in S, we have $\inf_\psi \langle\psi|F|\psi\rangle \geq 0$ and $\sup_\psi \langle\psi|F|\psi\rangle \leq 1$, where infimum and supremum are over all normalized vectors in $\mathbb{C}^\infty$. Thus, $0 \leq F \leq \mathbb{1}$, and hence F is self-adjoint.

Let $\zeta \in \mathbb{C}^\infty$ be an arbitrary normalized vector. If $\zeta \in S$ then, by construction, $\mathbf{f}(\zeta) = \langle\zeta|F|\zeta\rangle$. Now we want to show that this equation is also true if $\zeta \notin S$. In this case, define the sequence of subspaces $T_1 := \mathrm{span}\{\zeta\}$ and $T_{n+1} := \mathrm{span}\,(S_n \cup \{\zeta\})$ for all $n \in \mathbb{N}$. Clearly $\dim T_n = n$ and $\bar{T} = \mathbb{C}^\infty$ for $T = \bigcup_{n\in\mathbb{N}} T_n$. Thus, according to our intermediate claim, there is a bounded operator G such that $\mathbf{f}(\psi) = \langle\psi|G|\psi\rangle$ for all normalized $\psi \in T$; in particular, $\mathbf{f}(\zeta) = \langle\zeta|G|\zeta\rangle$. But since $S \subset T$, we also have $\mathbf{f}(\psi) = \langle\psi|G|\psi\rangle$ for all $\psi \in S$. But, according to our intermediate statement, F is the unique bounded operator satisfying this equation, hence $F = G$.

As a side remark, note that the operator sequence $F^{(n)}$ does not in general converge to F in operator norm. □

Theorem 21. Suppose that for each finite d all OPFs $\mathbf{f} \in \mathcal{F}_d$ are of the form (91). Then the "closedness under system composition" assumption (Definition 9) implies that all OPFs $\mathbf{f} \in \mathcal{F}_\infty$ are also of the form

$$\mathbf{f}(\psi) = \langle\psi|F|\psi\rangle \ , \tag{94}$$

where the $\mathbb{C}^\infty$-operator F satisfies $0 \leq F \leq \mathbb{1}$.

Proof. Let us fix a finite-dimensional subspace $S \subset \mathbb{C}^\infty$. Denote the dimension of S by d. Let us fix an orthonormal basis $\psi_1, \ldots, \psi_d$ of S, an orthonormal basis $\alpha_1, \ldots, \alpha_d$ of $\mathbb{C}^d$, and a normalized vector $\beta \in \mathbb{C}^\infty$.

The Hilbert spaces $\mathbb{C}^\infty$ and $\mathbb{C}^d \otimes \mathbb{C}^\infty$ are isomorphic in a very non-unique way; so let $X : \mathbb{C}^d \otimes \mathbb{C}^\infty \to \mathbb{C}^\infty$ be an isometry such that

$$X(\alpha_i \otimes \beta) = \psi_i \ , \tag{95}$$

for all $i = 1, \ldots, d$ (this does not determine X uniquely; we will pick any such X arbitrarily). Hence, for any vector $\psi \in S$ there is $\alpha \in \mathbb{C}^d$ such that $\psi = X(\alpha \otimes \beta)$. And for any OPF $\mathbf{f}$ of $\mathbb{C}^\infty$,the OPF $G := f \circ X$ must be well-defined, since $\mathcal{F}_\infty$ is closed under composition with unitaries. In particular,

$$\mathbf{f}(\psi) = \mathbf{f}(X(\alpha \otimes \beta)) = \mathbf{g}(\alpha \otimes \beta) \qquad \text{for all } \psi \in S. \tag{96}$$

Note that due to the mentioned isomorphism both, $\mathbf{f}$ and $\mathbf{g}$, belong to $\mathcal{F}_\infty$.

At this point we invoke "closedness under system composition" (Definition 9). This tells us that for any OPF $\mathbf{g} \in \mathcal{F}_\infty$ of $\mathbb{C}^d \otimes \mathbb{C}^\infty$ there is $\mathbf{h} \in \mathcal{F}_d$ such that

$$\mathbf{h}(\alpha) = \mathbf{g}(\alpha \otimes \beta) \ , \tag{97}$$

for all $\alpha \in \mathrm{P}\mathbb{C}^d$. This together with Corollary 19 implies that there is a $\mathbb{C}^d$-matrix H such that $0 \leq H \leq \mathbb{1}$ and $\mathbf{h}(\alpha) = \langle\alpha|H|\alpha\rangle$.

Next we decompose H in the chosen orthonormal basis of $\mathbb{C}^d$, obtaining $H = \sum_{i,j=1}^d h_{ij}|\alpha_i\rangle\langle\alpha_j|$. Also, we use the coefficients h_{ij} to define the $(\mathbb{C}^d \otimes \mathbb{C}^\infty)$-operator $F_S = \sum_{i,j=1}^d h_{ij}|\psi_i\rangle\langle\psi_j|$, which is supported on the subspace S and satisfies $0 \leq F_S \leq \mathbb{1}$.

Finally, for any given normalized $\psi \in S$, we decompose it in the chosen S-basis $\psi = \sum_{i=1}^d x_i\, \psi_i$; it follows that $\alpha = \sum_{i=1}^d x_i\, \alpha_i \in \mathbb{C}^d$. Combining this with (96) and (97) we obtain

$$\mathbf{f}(\psi) = g(\alpha \otimes \beta) = \mathbf{h}(\alpha) = \langle\alpha|H|\alpha\rangle = \sum_{ik} \bar{x}_i h_{ij} x_j = \langle\psi|F_S|\psi\rangle \ . \tag{98}$$

In summary, for any given finite-dimensional subspace S, we have constructed a $\mathbb{C}^\infty$-operator F_S satisfying the premises of Lemma 20. This gives us the conclusion of Theorem 21. □

Supplementary note 5: The post-measurement state-update Rule

Until now we have been concerned with the outcome probabilities of quantum measurements. In this section, we characterize the transformation that the quantum state undergoes during the measurement process.

Lemma 22 (quantum post-measurement state-update rule). The only post-measurement state-update rule compatible with the quantum probability assignment (91-92) is such that each measurement outcome is represented by a completely-positive linear map Λ. The probability of this outcome is given by

$$P(\Lambda|\psi) = \mathrm{tr}\Lambda(|\psi\rangle\langle\psi|) \ , \tag{99}$$

and the post-measurement state after outcome Λ is

$$\rho = \frac{\Lambda(|\psi\rangle\langle\psi|)}{\mathrm{tr}\Lambda(|\psi\rangle\langle\psi|)} \ . \tag{100}$$

In this statement each outcome is characterized by a map Λ, while in Corollary 19 each outcome is characterized by s POVM elements F. This two mathematical descriptions of an outcome are connected via

$$\mathrm{tr}\Lambda(|\psi\rangle\langle\psi|) = \langle\psi|F|\psi\rangle \ , \tag{101}$$

for all ψ.

The remainder of this section constitutes the proof of Lemma 22. While the mathematics of this proof is certainly not new, we give the details in terms of the context and formalism of this paper.

Proof. Corollary 19 states that any measurement has OPFs $\{\mathbf{f}_i\}$ of the form $\mathbf{f}_i(\psi) = \mathrm{tr}[F_i|\psi\rangle\langle\psi|]$, where $\{F_i\}$ are positive operators satisfying $\sum_i F_i = \mathbb{1}$, that is, a POVM. This implies that all the statistical information of any ensemble (ψ_r, p_r) is given by the corresponding density matrix $\rho = \sum_r p_r|\psi_r\rangle\langle\psi_r|$. The associated linear form $\Omega_\rho : \mathbb{C}\mathcal{F}_d \to \mathbb{C}$ is given by $\Omega_\rho(\mathbf{f}_i) = \mathrm{tr}(\rho F_i)$, relating the usual density matrix formalism to the general formalism of this paper.

At this point we still have not said anything about the post-measurement state update rule. But whatever this rule is, let $\sigma(F_i, \rho)$ be the post-measurement state (that is, its density matrix) after outcome F_i, when the initial state is ρ. And define the map Λ_{F_i} which takes the original state ρ to the post-measurement state times its corresponding probability:

$$\Lambda_{F_i}(\rho) := \sigma(F_i, \rho)\,\mathrm{tr}[F_i\rho] \ . \tag{102}$$

Next, consider another given measurement with POVM $\{G_j\}$, and define the POVM $\{H_{j,i}\}$ to be that corresponding to the successive implementation of the measurements $\{F_i\}$ and $\{G_j\}$. (This must correspond to a valid measurement, because the whole point of talking about a post-measurement state is that one can make further measurements on it.) Then, using the rules of probability calculus and the above formulas we obtain

$$\begin{aligned} \mathrm{tr}[H_{j,i}\rho] &= P(j,i) = P(j|i)P(i) = \mathrm{tr}[G_j\sigma(F_i,\rho)]\mathrm{tr}[F_i\rho] \\ &= \mathrm{tr}[G_j\Lambda_{F_i}(\rho)] \ , \end{aligned}$$

for all i, j and ρ. This equation implies that the map $\Lambda_{F_i}(\rho)$ is linear in ρ.

By definition, the map Λ_{F_i} takes every valid density matrix to a non-negative multiple of another valid density matrix, hence, the map Λ_{F_i} is positive and trace-non-increasing. To recover formulas (99) and (100) we use the fact that $\mathrm{tr}\,\sigma(F_i, \rho) = 1$, which gives $\mathrm{tr}\Lambda_{F_i}(\rho) = \mathrm{tr}[F_i\rho] = P(F_i|\rho)$. In summary, the probability of an outcome is the trace of the unnormalized post-measurement state given by the map Λ associated to the outcome F under consideration. This allows to fully characterize an outcome with the corresponding map Λ, with no reference to a POVM element F.

Finally, we show that each outcome map Λ is not just positive, but completely positive. As argued in the main text, we use the fact that one can always regard a system $\mathbb{C}^d$ as part of a larger system $\mathbb{C}^d \otimes \mathbb{C}^b$. Then, the outcome map Λ must remain a valid outcome map when extended to the larger system $\Lambda \otimes \mathcal{I}$, where $\mathcal{I}$ is the identity map on the Hermitian operators acting on $\mathbb{C}^b$. This is the definition of complete positivity. □

Supplementary note 6: Technical result

Lemma 23. The kernel of the partial-trace map

$$\mathrm{tr}_n : \mathcal{M}_n^d \to \mathcal{M}_{n-1}^d \ , \tag{103}$$

$$M \mapsto \mathrm{tr}_n M \ , \tag{104}$$

is the SU(d) irrep with Dynkin diagram

- $(2n)$ if $d = 2$ (also known as spin=n),
- $(n, \underbrace{0, \ldots, 0}_{d-3}, n)$ if $d \geq 3$.

We denote these family if irreps by $\mathcal{N}_n^d$.

Proof. First we note that the element $N_{n,n} = |0\rangle\langle 1|^{\otimes n} \in \mathcal{M}_n^d$ satisfies $\mathrm{tr}_n N_{n,n} = 0$, so it is contained in the kernel of the map (103). Also, we note that the element $N_{n,n}$ is the highest weight vector of the irrep $\mathcal{N}_n^d$ having the Dynkin diagram specified in the statement of this lemma. Hence the irrep $\mathcal{N}_n^d$ is contained in the kernel.

To complete the proof of this lemma we only need to show that $\mathcal{N}_n^d$ is the only irrep inside the kernel. This is equivalent to the dimensional matching

$$\mathrm{dim}\mathcal{M}_n^d = \mathrm{dim}\mathcal{N}_n^d + \mathrm{dim}\mathcal{M}_{n-1}^d \ , \tag{105}$$

implied by the Isomorphism Theorem. In order to check the above identity we use the dimensional formula given in page 224 of [2], which tells us

$$\mathrm{dim}\mathcal{M}_n^d = \binom{d+n-1}{n}^2 , \tag{106}$$

$$\mathrm{dim}\mathcal{N}_n^d = \left(\frac{2n}{d-1}+1\right)\prod_{k=1}^{d-2}\left(1+\frac{n}{k}\right)^2 . \tag{107}$$

With some calculation we get

$$\begin{aligned}
&\mathrm{dim}\mathcal{M}_n^d - \mathrm{dim}\mathcal{M}_{n-1}^d \\
&\quad = \binom{d+n-1}{n}^2 - \binom{d+n-2}{n-1}^2 \\
&\quad = \frac{(d+n-1)!^2}{(d-1)!^2\, n!^2} - \frac{(d+n-2)!^2}{(d-1)!^2 (n-1)!^2} \\
&\quad = \frac{(d+n-1)!^2 - n^2 (d+n-2)!^2}{(d-1)!^2\, n!^2} \\
&\quad = \frac{(d+n-2)!^2}{n!^2 (d-2)!^2}\ \frac{(d+n-1)^2 - n^2}{(d-1)^2} \\
&\quad = \frac{(d+n-2)!^2}{n!^2 (d-2)!^2}\ \frac{d-1+2n}{d-1} \ ,
\end{aligned} \tag{108}$$

and

$$\begin{aligned}
\mathrm{dim}\mathcal{N}_n^d &= \left(\frac{2n}{d-1}+1\right)\prod_{k=1}^{d-2}\left(1+\frac{n}{k}\right)^2 \\
&= \frac{2n+d-1}{d-1}\left[(1+n)\left(1+\frac{n}{2}\right)\cdots\left(1+\frac{n}{d-2}\right)\right]^2 \\
&= \frac{2n+d-1}{d-1}\left[\frac{n+1}{1}\frac{n+2}{2}\cdots\frac{n+d-2}{d-2}\right]^2 \\
&= \frac{(d+n-2)!^2}{n!^2 (d-2)!^2}\frac{2n+d-1}{d-1} \ .
\end{aligned} \tag{109}$$

This shows that the dimensional matching (105) holds. □

Supplementary discussion

In this section we compare the theorem presented in this work with recent work in the same direction.

Ref. [5] considers probability assignments (i.e. correlation tables) for sets of measurements and their exclusivity relations. It is shown that the *exclusivity principle* (derived from properties of ideal measurements), together with an assumption of composability of experiments, restricts those correlations to be exactly those allowed by quantum theory. However, this does not prove that outcome probabilities of measurements on quantum states must be given by the Born rule; states and unitaries do not play any role in [5]. This is a very different approach from the one in our paper. We do not assume the existence of ideal measurements, but show that the Born rule follows (under minimal operational assumptions) from the dynamical postulates of quantum theory.

In [6] the Born rule is recovered from postulates which are non-probabilistic and the assumption that measurement outcomes correspond to projectors. This is comparable to the decision theoretic approach of Deutsch [7] and Wallace [8] which also seeks to account for the existence of probabilities. This is in contrast to the present work, where we do not seek to explain the emergence of probabilities in quantum theory, nor do we associate measurement outcomes to projectors.

Supplementary References

[1] Galley, T. D. and Masanes, L. Classification of all alternatives to the Born rule in terms of informational properties. *Quantum* **1**, 15(2017).

[2] Fulton, W. and Harris, J. *Representation theory : a first course.* Graduate texts in mathematics. Springer-Verlag, New York, Berlin, Paris, (1991).

[3] Hardy, L. Quantum theory from five reasonable axioms. *Preprint at https://arxiv.org/abs/quant-ph/0101012* (2001).

[4] Goldberg, M. and Tadmor, E. On the numerical radius and its applications. *Linear Algebra and its Applications* **42**, 263 – 284(1982).

[5] Cabello, A. The physical origin of quantum nonlocality and contextuality. *Preprint at https://arxiv.org/abs/1801.06347* (2018).

[6] Frauchiger, D. and Renner, R. A non-probabilistic substitute for the Born rule. *Preprint at http://arxiv.org/abs/1710.05033* (2017).

[7] Deutsch, D. Quantum theory of probability and decisions. *Proceedings of the Royal Society of London A: Mathematical, Physical and Engineering Sciences* **455**(1988), 3129–3137(1999).

[8] Wallace, D. *Many Worlds? Everett, Quantum Theory, and Reality*, chapter How to Prove the Born Rule. Oxford University Press(2010).
